# Spatial-aware lightweight network for real-time tea disease detection: A coordinate attention-enhanced YOLOv8n approach with path-decoupling strategy

**DOI:** 10.1371/journal.pone.0354583

**Published:** 2026-07-24

**Authors:** Xiang Lyu, Yue Yu, ChengLei Song

**Affiliations:** 1 School of Information Engineering, Xinyang Agriculture and Forestry University, Xinyang, China; 2 School of Management, Xinyang Agriculture and Forestry University, Xinyang, China; Sichuan University, CHINA

## Abstract

The intelligent identification of tea diseases is crucial for ensuring tea quality and reducing economic losses in the tea industry. However, the deployment of deep learning models on edge devices remains challenging due to the conflict between detection accuracy and computational overhead. To address this, we propose CA-YOLOv8n, a lightweight object detection model tailored for tea disease diagnosis. Specifically, we introduce a Path-Decoupling strategy to streamline the network structure and integrate the Coordinate Attention (CA) mechanism to enhance the model's spatial awareness of subtle pathological features. Experimental results demonstrate that the proposed model achieves a mean Average Precision (mAP@50) of 98.89% while reducing the parameter count by 32.6% and FLOPs by 24.1% compared to the baseline YOLOv8n. The model was integrated into a diagnostic platform with an automated reporting interface, demonstrating that real-time tea disease identification is feasible on commodity CPU hardware in resource-constrained agricultural environments.

## 1. Introduction

The identification of plant phenotypes, particularly leaf diseases, has transitioned from traditional botanical taxonomy to automated computational analysis. In the early stages, research predominantly focused on manual feature engineering, where color histograms, texture descriptors (e.g., LBP), and geometric attributes were extracted to support shallow learning classifiers such as Support Vector Machines (SVM) and Random Forests [1]. While these methods provided a foundational framework for digital agriculture, their efficacy was often constrained by the high variance of field environments, such as fluctuating lighting and complex canopy backgrounds.

The subsequent emergence of Deep Learning (DL) has fundamentally shifted this paradigm. Convolutional Neural Networks (CNNs) have become the de facto standard for tea disease diagnosis due to their hierarchical feature extraction capabilities. Recent studies have demonstrated that architectures such as ResNet and DenseNet can achieve high classification accuracy under controlled conditions, marking a significant milestone in moving from laboratory-based research to potential field applications [2]. The rapid development of AI technology offers new possibilities for addressing the threat of pests and diseases to crops, especially through the design of intelligent early warning systems [3]. Currently, the academic focus has shifted from simple image classification to precise object detection and semantic segmentation. The You Only Look Once (YOLO) framework represents the mainstream direction in real-time diagnostic systems, prioritizing a balance between inference speed and detection precision. Soeb et al. utilized YOLOv7 to identify diverse tea diseases, supporting the feasibility of one-stage detectors in multi-class agricultural scenarios [4].

Despite substantial progress, several research gaps remain unresolved. First, there is an ongoing debate regarding the optimal balance between model depth and spatial fidelity. While deeper networks improve semantic understanding, they often suffer from “spatial information dilution” during repeated pooling operations, leading to significant false negative rates for subtle lesions, such as early-stage White spot. Second, traditional attention mechanisms, notably Squeeze-and-Excitation (SE), often overlook critical positional encoding by globally compressing spatial features. As demonstrated in research on high-density small target pest identification, embedding Coordinate Attention (CA) modules can significantly bolster the detection of small objects and help manage species diversity [5]. This lack of spatial awareness is detrimental when locating small and irregularly shaped lesions on tea leaves, which are often suppressed as noise in standard lightweight architectures [6]. Recent reviews underscore that state-of-the-art deep learning models have achieved accuracies, with detection and segmentation networks demonstrating precision rates above 90% in identifying complex agricultural infestations [7]. Furthermore, the lack of interpretable and architectures (Params < 5M) capable of maintaining high recall rates remains a significant barrier to the “on-site” deployment of intelligent tea plantation management tools. Achieving a precise balance between accuracy and efficiency is crucial for practitioners in fields like agriculture and forestry to make informed decisions on pest control [8].

Given the economic volatility of the tea industry and the increasing prevalence of climate-induced pest outbreaks, developing highly efficient diagnostic tools is of significant academic and practical urgency. This study addresses the aforementioned gaps by proposing CA-YOLOv8n, a framework that integrates Coordinate Attention (CA) to resolve the loss of spatial dependencies in lightweight networks. Unlike channel-based attention, CA embeds positional information into channel attention, capturing both direction-aware and position-sensitive dependencies. This enables the model to accurately pinpoint subtle pathological areas even in complex field backgrounds. The primary contributions of this work are threefold:

1. We propose CA-YOLOv8n, a lightweight detection model that achieves a mAP@50 of 98.89% while maintaining parameter scale.
2. We introduce a Path-Decoupling strategy to streamline the feature fusion network, reducing computational overhead without compromising the detection of multi-scale tea diseases.
3. We demonstrate edge-deployment feasibility on a diagnostic platform, achieving a + 1.21 percentage points (pp) improvement in Recall and a 23.2% reduction in CPU inference latency over the baseline, thus offering a viable technical route for real-time on-site tea plantation monitoring.

## 2. Related work

### 2.1 Evolution and taxonomy of agricultural disease monitoring

The identification of plant phenotypes has evolved significantly from traditional digital image processing to advanced deep representation learning. Historically, textural analysis via Gray-Level Co-occurrence Matrices (GLCM) and Local Binary Patterns (LBP) established the foundation for early automated diagnosis [9]. In particular, the evolution from traditional machine learning to Vision Transformers and hybrid models reflects a growing demand for models that can handle complex data-processing tasks in smart agriculture [10]. However, as reviewed by Wu et al., the integration of multimodal data fusion and dynamic prediction is driving a shift toward proactive pest management [2]. Recent research highlights a clear taxonomy in diagnostic methodologies, ranging from hyperspectral imaging and non-visualization techniques to modified deep learning architectures [11]. While early CNN-based models like AlexNet and Inception-ResNet demonstrated high accuracy in controlled environments, their robustness often diminishes under the “harsh environments” of real tea plantations, characterized by leaf shading and non-uniform illumination [12,13].

### 2.2 Object detection frameworks: Balancing precision and efficiency

The development of object detection for agricultural tasks is broadly categorized into region-based and regression-based frameworks. While two-stage detectors such as Faster R-CNN prioritize localization precision, their inherent computational latency remains a barrier for on-site diagnostic tasks [14]. Consequently, one-stage detectors, particularly the YOLO (You Only Look Once) series, have gained dominance due to their superior inference speed. Nayar et al. demonstrated that YOLOv8 achieves higher precision and faster detection compared to earlier versions on complex plant datasets [15]. Furthermore, Wang et al. optimized the YOLOv8 structure with Global Attention Mechanisms (GAM) to enhance the weights of critical feature information [16]. In the specific domain of rice and tea, Anitha et al. and Deng et al. validated that the iterative versions of YOLO (up to YOLOv10s) provide an efficient technical route for multi-class identification in unstructured backgrounds [17,18]. Comparative evaluations of state-of-the-art techniques consistently demonstrate the superiority of modern AI-based approaches, which often outperform older image analysis methods in both speed and accuracy.

### 2.3 Lightweight design and edge computing deployment

To bridge the gap between laboratory algorithms and field-based applications, researchers have prioritized model lightweighting. Guan et al. designed “Dise-Efficient,” a compact architecture (13.3 MB) that maintains high accuracy while facilitating deployment on embedded devices [19]. Similarly, Zhao et al. proposed M2CNet, a hierarchical pyramid structure that uses multi-head attention to reduce parameters to as low as 1.8M [20]. In the context of tea plantations where connectivity is often limited, edge computing paradigms offer a tractable model for mainstreaming smart agriculture [21]. Intelligent detection models integrating Transformer technology and knowledge graphs have shown promising results in edge computing solutions, ensuring rapid response capabilities on mobile platforms [22]. Recent studies focus on optimizing models for specific hardware; for instance, Aha et al. and Khan et al. demonstrated that post-training quantization of MobileNetV3 can reduce parameters significantly without compromising classification integrity, enabling real-time diagnostic performance on mobile platforms [23,24].

### 2.4 Diversified applications of visual attention mechanisms

To recover spatial information lost during downsampling, various attention mechanisms have been integrated into agricultural detectors. For instance, enhanced models for corn pest recognition have successfully addressed network degradation by introducing additional effective channels, facilitating the extraction of crucial deep features [25]. While Squeeze-and-Excitation (SE) recalibrates channel importance, it neglects positional encoding, leading to potential leakage in detecting small lesions [26]. To address this, Qian et al. proposed a Mixed Attention Module (MAM) to model long-range dependencies within the channel domain while extracting fine-grained features [27]. Furthermore, Xue et al. developed “YOLO-Tea,” which integrates Convolutional Block Attention Module (CBAM) to allow models to better focus on shaded disease spots [28]. Jia et al. and Li et al. specifically emphasized that Coordinate Attention (CA) embeds positional information into channel maps, which is vital for distinguishing irregular morphologies of tea pests from the visual noise of the tea canopy [29,30]. These advancements provide the theoretical impetus for the CA-integrated framework evaluated in this study. Table 1 presents a comparison of the discussed models for agricultural disease detection.

**Table 1:** Comparison of existing models for agricultural disease detection.

Model	Base Architecture	Key Enhancements	Limitations
YOLO-Tea (2023) [28]	YOLOv5	ACmix, CBAM, RFB, GCNet	Older YOLOv5 baseline with relatively high parameter overhead
YOLOv8-RCAA (2024) [31]	YOLOv8	RepVGG, CBAM, Anchor-free, ATSS	Structural re-parameterization increases training complexity and memory usage
MobileNet-CA-YOLO (2023) [29]	YOLOv7 + MobileNetV3 backbone	Coordinate Attention (CA) on backbone	CA applied to backbone only, without neck simplification; trained on rice pest data, not tea
Dise-Efficient (2023) [19]	EfficientNetV2	Dynamic LR decay, Transfer learning	Reported accuracy drop in real-world complex environments
AX-RetinaNet (2022) [32]	RetinaNet	Multiscale feature fusion, Channel Attention	Heavyweight anchor-based architecture incurs high computational latency

## 3. Methodology

### 3.1 Dataset acquisition and description

The dataset is hosted on figshare and comprises 9,591 tea-leaf images across eight categories: Black rot of tea, Brown blight of tea, Leaf rust of tea, Red Spider infested tea leaf, Tea Mosquito bug infested leaf, healthy Tea leaf, White spot of tea, and a residual disease class covering minor pathologies outside the seven specific categories above. The images were originally compiled from publicly accessible tea-disease pages on the Tencent Developer Community and processed through the Roboflow data-preparation pipeline; the figshare deposit re-hosts the bundle in YOLO format with the train/valid/test split folders and the data.yaml descriptor. Of the 9,591 images, 8,044 are allocated to training and validation and the remaining 1,547 (~16%) form an independent test set used for final evaluation. Mosaic augmentation is applied during training to mitigate spatial over-fitting and reinforce sensitivity to localised lesions.

#### 3.1.1 Statistical distribution.

Of the 9,591 images in the dataset, the standard split allocates 7,260 images to training, 784 to validation, and 1,547 to independent testing (approximately 16% of the total). The per-class distribution of bounding-box instances is highly imbalanced and spans more than three orders of magnitude. Tea Mosquito bug contributes the largest count (7,254 total instances), followed by Leaf rust (3,645), Red Spider (1,022), Tea leaf (healthy, 427), White spot (324), and Black rot (128). At the rare end of the distribution, Brown blight comprises only 8 instances in total (4 in training, 0 in validation, 4 in test), and the residual disease aggregate class contains only 4 training instances with no representation in the validation or test splits. This severe long-tailed distribution has direct downstream consequences for the ablation analysis: in particular, the working-point instability observed for the + CA-only variant on Brown blight (Section 4.4 and Fig. 9b) is consistent with the very small training-instance count for this class (n = 4), which leaves the optimal confidence threshold highly sensitive to architectural perturbations; the addition of Path-Decoupling in the full model stabilizes the operating point and restores full Recall. The complete absence of disease instances in the test split, together with the semantic vagueness of this residual aggregate label, motivates omitting this class from per-class results (see Fig. 10 caption) and is acknowledged as a study limitation.

**Fig 1:**
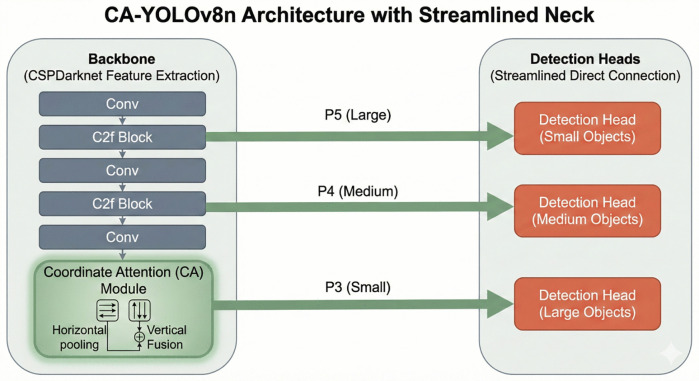
CA-YOLOv8n architecture.

**Fig 2:**
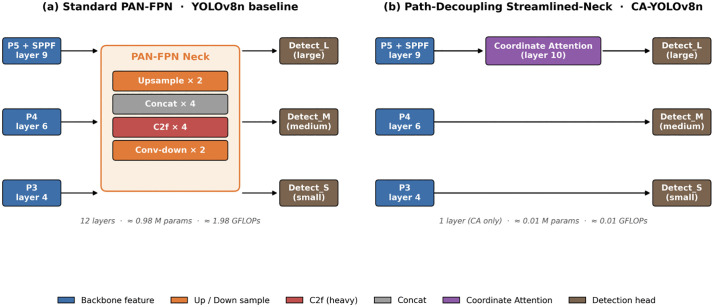
Architectural comparison between (a) the standard PAN-FPN neck used in YOLOv8n and (b) the proposed Path-Decoupling Streamlined-Neck adopted in CA-YOLOv8n.

**Fig 3:**
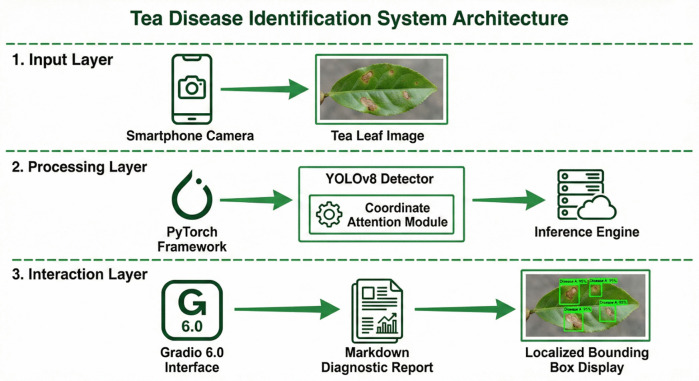
Architectural framework of the tea disease identification system based on CA-YOLOv8n.

**Fig 4:**
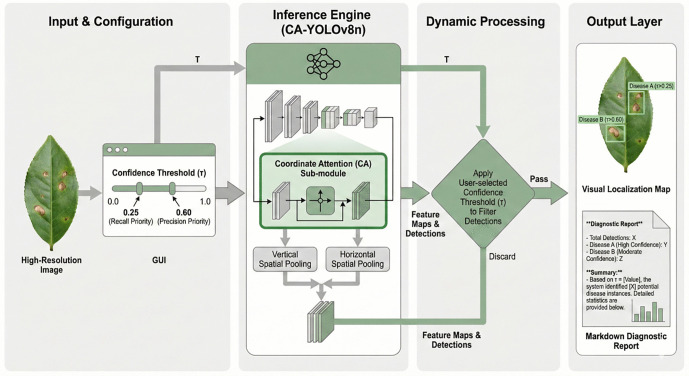
Detailed inference pipeline and dynamic threshold decision-making logic based on CA-YOLOv8n.

**Fig 5:**
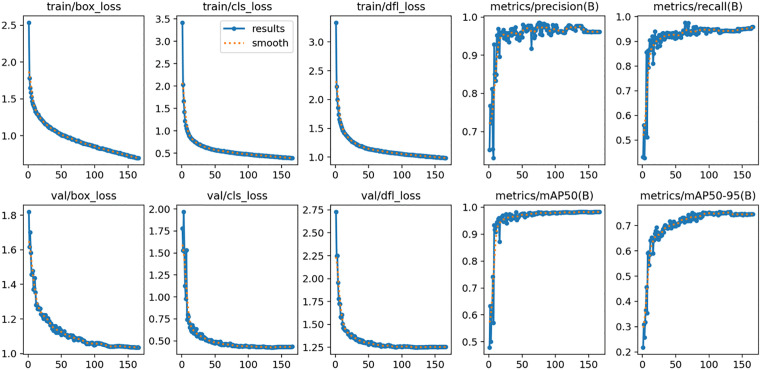
Training and validation loss curves (Box, Class, and DFL losses) of CA-YOLOv8n.

**Fig 6:**
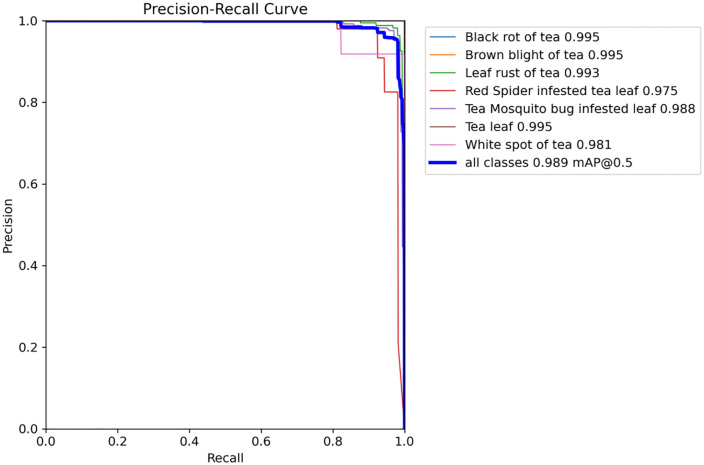
Precision-Recall (P-R) curves on the test dataset.

**Fig 7:**
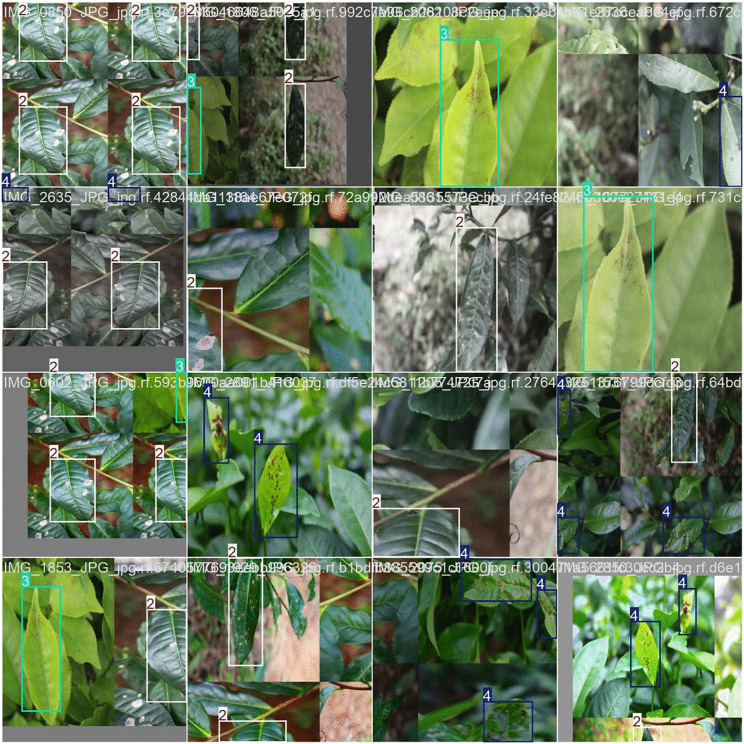
Batch samples. (Note: The sample images in this figure are sourced from our tea-leaf disease dataset deposited at figshare, https://doi.org/10.6084/m9.figshare.32253357, available under a CC BY 4.0 license).

**Fig 8:**
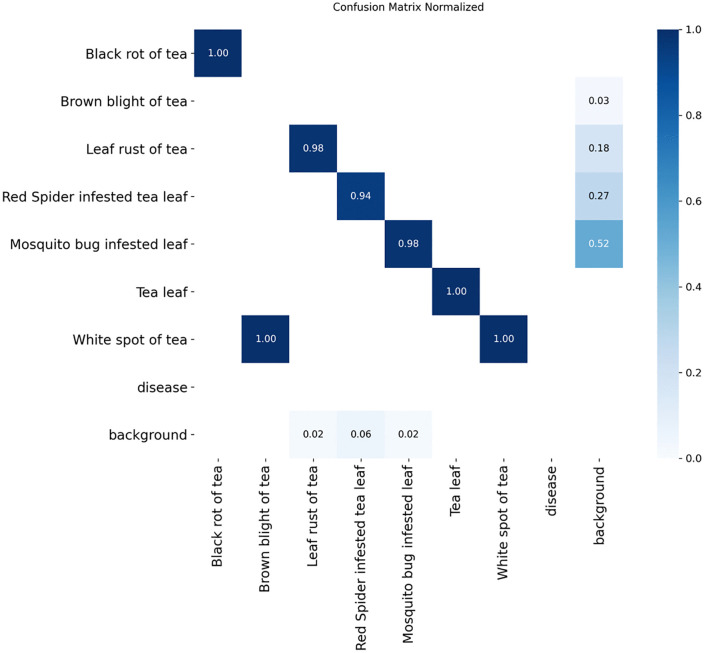
Confusion matrix of the CA-YOLOv8n model evaluated on the independent test set.

### 3.2 Overall framework of CA-YOLOv8n

The identification of tea diseases in natural environments requires a model that balances an ultra-lightweight design with high spatial sensitivity. This study proposes CA-YOLOv8n, an architectural refinement optimized for edge-side agricultural deployment. The core innovation lies in the Path-Decoupling strategy, which streamlines the redundant PAN-FPN neck structure into a Streamlined-Neck. Unlike the standard YOLOv8n, our model bypasses iterative feature aggregation layers and directly routes the P3, P4, and P5 feature maps from the backbone to the detection heads. To mitigate the potential loss of multi-scale context, we strategically integrate the Coordinate Attention (CA) mechanism at the terminal of the backbone. This “Less-is-More” philosophy minimizes parameters to 2.03M while ensuring that incipient lesions remain distinguishable against complex canopy backgrounds. The detailed architectural configuration and the specific integration of the CA module within the modified C2f block are illustrated in Fig 1.

The standard neck stacks twelve fusion operations (2 Upsample + 4 Concat + 4 C2f + 2 Conv-down. The Path-Decoupling design retains only a single Coordinate Attention module on the deepest backbone feature (P5) and routes the P3 and P4 features directly to their corresponding detection heads, eliminating both the FPN top-down and the PAN bottom-up fusion paths. Architectural comparison between the standard PAN-FPN neck used in YOLOv8n and the proposed Path-Decoupling Streamlined-Neck adopted in CA-YOLOv8n in Fig 2.

### 3.3 Coordinate attention and architectural compensation

Standard channel attention mechanisms (e.g., SE) overlook positional encoding, which is vital for localizing micro-scale lesions. In CA-YOLOv8n, the CA module acts as a spatial compensator for the Streamlined-Neck. By factorizing 2D global pooling into two 1D feature encoding processes, it maintains high localization rigor despite the reduced depth of the neck. The two 1-D pooling kernels of size (H, 1) and (1, W) aggregate features along the vertical and horizontal axes respectively; the resulting pair of direction-aware descriptors are concatenated, passed through a shared 1 × 1 convolution, and split back into a height-wise and a width-wise attention map. Each map is broadcast over its orthogonal axis when modulating the input, so the recalibration weights vary across spatial coordinates rather than being shared within a channel. This row- and column-conditioned gating, which encodes both channel saliency and the location along each axis at which it arises, is the spatial-aware characteristic that distinguishes CA from the position-agnostic global gating of SE [33] and its channel-only variants.

The factorisation matches the visual structure of tea-leaf disease imagery, where lesions are typically small, irregularly scattered, and often elongated along leaf veins or clustered near leaf margins — directional cues that channel-only attention discards but that the row–column decomposition preserves. The two 1-D poolings also carry a lower computational footprint than full 2-D self-attention, which is consistent with the lightweight design goal of CA-YOLOv8n. The two stages of CA — Coordinate Information Embedding and Coordinate Attention Generation — are formalised below, followed by their integration under the Path-Decoupling design shown in Fig 2.

#### 3.3.1 Coordinate information embedding.

For an input X, CA utilizes two spatial kernels (H,1) and (1,W)to encode features. The output for the c−th channel at height h and width w is:


$$\begin{array}{c} {𝐳}_{𝐜}^{𝐡}(𝐡)=\frac{1}{𝐖}\sum_{0\leq 𝐢<𝐖}{𝐱}_{𝐜}\left(𝐡,𝐢\right) \end{array}$$ (1)

$$\begin{array}{c} {𝐳}_{𝐜}^{𝐰}(𝐰)=\frac{1}{𝐇}\sum_{0\leq 𝐣<𝐇}{𝐱}_{𝐜}\left(𝐣,𝐰\right) \end{array}$$ (2)

#### 3.3.2 Coordinate attention generation.

The two directional feature maps are concatenated and passed through a shared 1×1 convolutional transform $F_{\text{1}}$, followed by a non-linear activation function δ (h-swish) to produce an intermediate feature map f:


$$\begin{array}{c} 𝐟=\delta ({𝐅}_{1}([{𝐳}_{𝐡},{𝐳}_{𝐰}]))\in {𝐑}^{𝐂/𝐫\times (𝐇+𝐖)} \end{array}$$ (3)

where r is the reduction ratio used to control the module size. Then,fis split into two separate tensors $f^{\text{h}}\in {\text{R}}^{C/r\times H}$ and $f^{w}\in {\text{R}}^{C/r\times W}$. Two additional 1×1 convolutions $F_{\text{h}}andF_{w}$ transform these into attention maps $g^{\text{h}}$ and $g^{w}$ using the


$$\begin{array}{c} {𝐠}^{𝐡}=\sigma ({𝐅}_{𝐡}({𝐟}^{𝐡})) \end{array}$$ (4)

$$\begin{array}{c} {𝐠}^{𝐰}=\sigma ({𝐅}_{𝐰}({𝐟}^{𝐰})) \end{array}$$ (5)

#### 3.3.3 Feature recalibration under path-decoupling.

In our Streamlined-Neck architecture, this recalibration is critical. By applying the weights $g^{\text{h}}$ and $g^{w}$, the input feature map x is reweighted to produce the output y. This process embeds directional information into the latent representation, allowing the model to “anchor” the precise coordinates of tea lesions even without the iterative multi-scale fusion of a standard PANet:


$$\begin{array}{c} {𝐲}_{𝐜}(𝐡,𝐰)=\;{𝐱}_{𝐜}(𝐡,𝐰)\times {𝐠}_{𝐜}^{𝐡}(𝐡)\times {𝐠}_{𝐜}^{𝐰}\left(𝐰\right) \end{array}$$ (6)

### 3.4 Algorithm description

The training and inference logic of the CA-YOLOv8n system, optimized for Path-Decoupling, is summarized in Algorithm 1. The core of this approach is to transform the feature extraction process into a spatially-aware, streamlined operation. By embedding the Coordinate Attention (CA) module at the output of the backbone, the system factorizes the global spatial information into direction-aware feature maps.

Unlike the standard YOLOv8n, the proposed algorithm bypasses the intermediate FPN-PAN fusion stages, instead routing the CA-recalibrated multi-scale features (P3, P4, P5) directly to the decoupled detection heads. This logic ensures that the spatial geometry of irregular lesions—characteristic of pathologies like *Tea Mosquito bug* and *Leaf rust*—is emphasized without the risk of feature dilution during repetitive convolutions in the neck.

**Algorithm 1. CA-YOLOv8n training and inference logic with Path-Decoupling**

**Input:** Image $I\;\in \;R^{\text{6}\text{4}\text{0}\times \text{6}\text{4}\text{0}\times \text{3}}$, pretrained weights $W_{\text{pre}}$,

confidence threshold $\tau_{\text{conf}}\;=\;\text{0}.\text{2}\text{5}$, NMS threshold $\tau_{\text{nms}}\;=\;\text{0}.\text{4}\text{5}$

**Output:** Optimized bounding boxes B, class labels L, confidence scores S

***Stage 1: Feature extraction and spatial recalibration***

1 Initialize CA-YOLOv8n backbone with modified C2f blocks;

2 **for** each input batch I **do**

3 Extract multi-scale features through backbone layers $L_{\text{0}}\;\ldots \;L_{\text{9}}$;

4 At Layer 10, execute the Coordinate Attention (CA) mechanism;

5 Factorize 2D pooling into 1D horizontal and vertical encoding via Eq (1) & (2);

6 Generate direction-aware attention maps $g^{h}$ and $g^{w}$ via Eq (4) & (5);

7 $X_{\text{recalibrated}}\;\leftarrow \;X\;\times \;g^{h}\;\times \;g^{w}$; *// feature recalibration*

8 **end**

***Stage 2: Streamlined-Neck direct routing (architectural innovation)***

9 Extract recalibrated feature maps $P_{\text{3}},\;P_{\text{4}},\;P_{\text{5}}$ directly from the CA-augmented backbone;

10 Bypass the standard PAN-FPN neck to eliminate parameter redundancy;

11 Route $\left\{P_{\text{3}},\;P_{\text{4}},\;P_{\text{5}}\right\}$ directly to the decoupled detection heads;

***Stage 3: Inference and refinement***

12 Compute raw predictions for category L and bounding-box regression B;

13 Apply Non-Maximum Suppression (NMS) with $\tau_{\text{nms}}$ to suppress redundant candidates;

14 Filter results where $S\;<\;\tau_{\text{conf}}$;

15 **return** final detection set {B, L, S};

### 3.5 Training setup and hardware environment

Training configuration. All models reported in this paper—the CA-YOLOv8n full model, the baseline YOLOv8n, and the variants used in the ablation study (Section 4.4)—were trained under identical hyperparameters to ensure controlled comparison. Models were initialized from the official YOLOv8n pretrained weights and fine-tuned on the tea-disease dataset for up to 150 epochs with early stopping (patience = 30). The input resolution was fixed at 640 × 640 with a batch size of 16. Optimization followed the Ultralytics auto-policy with initial learning rate lr₀ = 0.01, final-lr factor 0.01, momentum 0.937, weight decay 5 × 10 ⁻ ⁴, and a 3-epoch warmup. Loss weights were set to box = 7.5, cls = 0.5, dfl = 1.5. Mosaic augmentation was applied throughout training and disabled for the final 10 epochs (close_mosaic = 10). Additional augmentations included horizontal flip (p = 0.5), HSV jittering (h = 0.015, s = 0.7, v = 0.4), random scaling (±0.5), translation (±0.1), random erasing (p = 0.4), and RandAugment. A fixed random seed (seed = 0) and deterministic mode were enabled to ensure reproducibility.

Hardware environment. Training was conducted on the Kaggle cloud platform using two NVIDIA Tesla T4 GPUs (16 GB each) in data-parallel mode, with PyTorch and the Ultralytics framework. To rigorously evaluate the model's edge-deployment feasibility, all inference-speed measurements reported in this paper were conducted on a consumer-grade CPU—a 13th-Gen Intel Core i5-13500H—without any GPU acceleration, TensorRT/ONNX conversion, or quantization-based optimization. This deliberate separation between the resource-intensive training environment and the lightweight on-site deployment environment confirms that CA-YOLOv8n can sustain real-time throughput on commodity hardware typical of tea-plantation field operations.

### 3.6 Experimental evaluation metrics

#### 3.6.1 Global precision and detection effectiveness.

To evaluate the fundamental identification capability, we employ mAP@50 and F1-score.


$$\begin{array}{c} 𝐅1-𝐬𝐜𝐨𝐫𝐞=2\times \frac{𝐏𝐫𝐞𝐜𝐢𝐬𝐢𝐨𝐧\times 𝐑𝐞𝐜𝐚𝐥𝐥}{𝐏𝐫𝐞𝐜𝐢𝐬𝐢𝐨𝐧+𝐑𝐞𝐜𝐚𝐥𝐥} \end{array}$$ （7）

#### 3.6.2 Spatial localization rigor.

A key highlight of this study is the integration of the CA mechanism. To demonstrate its superiority in precise lesion boundary regression, we utilize mAP@50–95. UnlikemAP@50, this metric averages performance across 10 IoU thresholds, reflecting the model's robustness in aligning predicted boxes with the actual edges of tea lesions, validating the spatial anchoring capability of the Coordinate Attention module.

#### 3.6.3 Edge computing gain ratio.

To quantify the trade-off between detection performance and computational footprint, we report the Precision-to-Parameter Ratio: PPR = mAP@50 (%) / Parameters (M). Higher PPR indicates a more parameter-efficient model. Latency on commodity CPU hardware is reported separately in Tables 2 and 3.

**Table 2:** Comparative performance on the tea disease test dataset.

Model	mAP@50(%)	Recall (%)	mAP@50–95 (%)	F1-score	Latency (ms)	FPS
YOLOv8n	97.11	97.41	74.42	0.923	63.62	15.7
YOLOv10n	98.86	81.86	75.8	0.889	179	5.6
CA-YOLOv8n	98.89	98.62	76.26	0.966	51.39	19.5

**Table 3:** Analysis of computational efficiency and parameter utilization.

Model	Parameters (M)	GFLOPs	PPR
YOLOv8n	3.01	8.20	32.26
YOLOv10n	8.07	12.4	12.25
CA-YOLOv8n	2.03	6.22	48.71

### 3.7 System implementation and deployment

#### 3.7.1 System software architecture.

The system architecture follows a high-performance modular design tailored for real-time agricultural diagnostics. The backend is constructed using PyTorch and the Ultralytics library, encapsulating the customized CoordAtt and h_swish classes to maintain mathematical consistency with the proposed spatial-encoding logic. For the user interface, the framework integrates Gradio 6.0, facilitating an asynchronous data pipeline from raw image acquisition (via numpy arrays) to Markdown-formatted diagnostic reporting. The software environment ensures model persistence through  .pt and ONNX formats, allowing the 2.03M parameter model to execute efficient inference on common hardware without extensive computational overhead. Identify the overall architecture of the system, as shown in Fig 3.

#### 3.7.2 Inference logic and model integration.

The system's core inference is encapsulated in the predict_tea function. Upon image acquisition, the backend loads the CA-YOLOv8n weights (best.pt) and executes a single forward pass. A default confidence threshold of τ=0.25 is applied to prioritise Recall over Precision, since missed lesions are more consequential than false positives in early-stage disease screening. A sensitivity sweep over τ∈[0.05, 0.60] shows the F1-score remaining within a stable region around 0.953; the 0.25 setpoint sits inside this stable region and is therefore selected as the operational threshold. False Negatives (FN) are thereby minimised — a critical requirement for early-stage disease prevention in tea plantations.

The inference pipeline follows a modular design: 1. Preprocessing: Standardizing input tensors for PyTorch compatibility. 2. Feature Extraction: Leveraging Coordinate Attention (CA) to recalibrate spatial features, ensuring precise localization even at low confidence levels. 3. Post-processing & Dynamic Adaptation: Parsing output tensors into class probabilities and rendering bounding boxes via OpenCV. The system provides a dynamic parameter interface, allowing users to adjust τ (e.g., increasing it to 0.60) to favor precision for late-stage automated statistics or decreasing it for high-sensitivity early screening. As shown in Fig 4, a detailed inference pipeline and dynamic threshold decision-making logic based on CA-YOLOv8n.

#### 3.7.3 Interactive interface and multi-mode diagnostics.

The system's user interface is constructed using the Gradio framework, providing a seamless bridge between complex deep learning inference and practical agricultural applications. The interface is partitioned into three functional zones:

Strategic Parameter Control: Unlike traditional static systems, our interface features a Dynamic Confidence Slider. This allows researchers to toggle between “Screening Mode” (τ=0.25) for maximum disease recall and “Statistical Mode” (τ≥0.60) for high-precision automated reporting.

Visual Diagnostic Feedback: Upon execution, the system renders a high-definition localization map using OpenCV. The Coordinate Attention (CA)-enhanced bounding boxes are displayed with real-time confidence scores, providing visual verification of the model's spatial recalibration.

Comprehensive Statistical Summary: A dedicated Markdown module generates an instantaneous Diagnostic Report, aggregating lesion counts by category and presenting class-wise peak confidence scores. This dual-output (visual + quantitative) ensures that the diagnostic results are both interpretable and actionable for end-users.

#### 3.7.4 Performance advantages of lightweight deployment.

Leveraging the 2.03M parameter architecture, the system demonstrates high inference efficiency on edge platforms. The integration of CoordAtt ensures a low false-negative rate in complex tea plantation backgrounds characterized by canopy shadows. This implementation proves that the proposed Path-Decoupling modifications effectively resolve the “precision-efficiency trade-off,” offering a robust digital tool for sustainable crop protection.

## 4 Results and discussion

### 4.1 Monitoring of training process and convergence analysis

As illustrated in Fig 5, the three objective functions—Box Loss, Class Loss, and DFL Loss—exhibited a rapid, logarithmic decline during the first 40 epochs. The model reached convergence well before the 150-epoch upper bound, with the early-stopping criterion (patience = 30) terminating training once no further improvement in validation mAP was observed.

To evaluate the ultimate diagnostic efficacy of the converged model, the Precision-Recall (P-R) Curve was generated on the test set, as shown in Fig 6. The P-R curve provides a visualization of the trade-off between precision and recall across all tea disease categories. The aggregate mAP@50 reaching 98.89%.

This accelerated convergence and the high AUC (Area Under Curve) serve as empirical evidence that the Coordinate Attention (CA) module provides feature anchoring, allowing the optimizer to identify optimal weight configurations more efficiently than the baseline architecture. Furthermore, the high degree of synchronization between the training and validation curves, coupled with the robust P-R performance, confirms the effectiveness of Mosaic augmentation and the structural robustness of our modified backbone. These results ensure that the model has been fully optimized without redundant iterations, providing a high-performance weight foundation for field deployment.

To further ensure the model's robustness against complex plantation environments, Mosaic data augmentation was employed during the training phase. As shown in Fig 7, the training batch samples illustrate the stochastic synthesis of multiple images, which forces the model to learn localized pathological features even under partial occlusion and varying scales. This strategy significantly mitigates the risk of spatial over-fitting and provides a strong empirical basis for the high recall rates observed in subsequent testing.

### 4.2 Confusion matrix and classification detailed analysis

A normalised confusion matrix on the held-out test split is shown in Fig 8. Per-class True-Positive rates are 1.00 for Black rot of tea, Tea leaf (healthy), and White spot of tea; 0.98 for both Leaf rust of tea and Tea Mosquito bug infested leaf; and 0.94 for Red Spider infested tea leaf — all consistent with the aggregate Recall of 0.986 reported in Table 2. The Brown blight of tea and disease rows show no detectable diagonal mass, consistent with their negligible test support (see Section 3.1.1). Off-diagonal entries between the seven specific disease classes are at or near zero (for example, 0% confusion between Red Spider and Leaf rust), indicating that the model rarely confuses one disease type for another once a lesion is detected.

A separate pattern is visible in the background column of Fig 8. Of regions labelled as background in the ground truth, 52% are wrongly predicted as Tea Mosquito bug, 27% as Red Spider, 18% as Leaf rust, and 3% as Brown blight. This distribution mirrors the per-class instance counts described in Section 3.1.1: the model is biased toward the dominant training classes when forced to predict on visually ambiguous background. The proposed network therefore preserves Recall on true lesions (≥ 0.94 across the supported classes) but inherits the class-imbalance bias of the training distribution as a false-positive cost on noisy background regions — a trade-off acknowledged in Section 5.2.

Note on confusion-matrix entries versus per-class operating-point Recall. Because Fig 7 is rendered using the Ultralytics validator's default thresholds (conf = 0.25, IoU = 0.45), the off-diagonal mass routed to the “background” column reflects a strict-threshold view of detection. The corresponding values for Tea Mosquito bug (0.52) and Red Spider (0.27) therefore quantify the proportion of ground-truth instances mapped to background under this rigid operating point. By contrast, the per-class operating-point Recall reported elsewhere in this paper (Fig 10b, Section 4.4) is computed at the maximum-F1 confidence threshold and reaches 0.980 and 0.943 for the same two classes in the full CA-YOLOv8n model—values that exceed the strict-threshold operating points implied by the confusion matrix by approximately 50 pp and 21 pp, respectively. The two views are mutually consistent under the underlying precision–recall curve: the lesions are detectable with high confidence, but their optimal confidence threshold is class-dependent. This sensitivity motivates the threshold-policy design discussed in Section 3.7.2 (Inference Logic and Model Integration), where a Recall-favoring default (τ = 0.25) is paired with a user-adjustable slider for high-precision reporting modes.

### 4.3 Performance comparison and visualization analysis

#### 4.3.1 Visualization and qualitative comparison.

**Fig 9:**
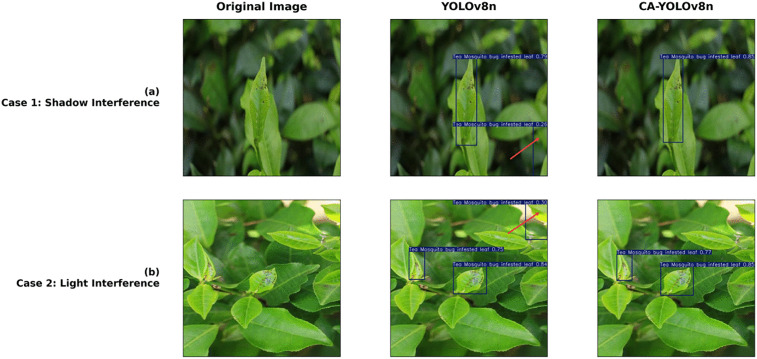
Qualitative comparison of detection results between baseline YOLOv8n and the proposed CA-YOLOv8n under complex tea plantation environments. (Note: The original tea leaf images used in this qualitative comparison are sourced from our dataset deposited at figshare, https://doi.org/10.6084/m9.figshare.32253357, available under a CC BY 4.0 license.).

To qualitatively evaluate the diagnostic robustness of the proposed CA-YOLOv8n, we performed a comparative visualization analysis against the baseline YOLOv8n under typical tea plantation challenges. Fig 9 illustrates the detection performance in two representative scenarios: shadow-induced false positives and light-interference misidentifications.

As illustrated in Fig 9(a) (Case 1: Shadow Interference), the baseline YOLOv8n exhibits vulnerability to complex background textures. In the shaded regions of the canopy, the baseline model misidentified a dark leaf edge and background shadows as “Tea Mosquito bug” lesions, resulting in an obvious False Positive. Fig 9(b) (Case 2: Light Interference) demonstrates the models’ performance under intense natural illumination. The baseline model produced a misidentification on a highly reflective leaf tip in the upper-right corner. Furthermore, the localization of existing lesions was slightly redundant.

These comparative results indicate that the proposed CA-YOLOv8n possesses superior feature discrimination capabilities in unstructured environments. By integrating the Coordinate Attention (CA) mechanism, the model is able to encode direction-aware spatial information, which helps distinguish between pathological textures and environmental noise such as shadows or specular reflections. Furthermore, the Path-Decoupling strategy effectively streamlines the feature flow, reducing the redundancy observed in the baseline model. Consequently, the improved model demonstrates higher diagnostic reliability and localization precision, effectively mitigating the common “over-detection” issues prevalent in standard YOLOv8n when deployed in real-world tea plantations.

#### 4.3.2 Quantitative metric evaluation.

The performance of both models on the independent test set (1,547 images) was quantified using standard evaluation metrics. The results are presented in Table 2.

Latency/FPS measured on a 13th-Gen Intel Core i5-13500H CPU at 640 × 640, batch = 1, FP32. According to Table 2, CA-YOLOv8n improves overall detection performance over the baseline YOLOv8n while simultaneously reducing the computational footprint. The mAP@50 increases from 97.11% to 98.89% (+1.78 pp), and Recall rises from 0.974 to 0.986 (+1.21 pp), reducing missed detections in early-stage screening. The mAP@50–95 metric—which evaluates bounding-box localization across stricter IoU thresholds—improves by 1.84 pp (74.42% → 76.26%), and the F1-score rises from 0.923 to 0.966, indicating that the gain in Recall is not offset by a Precision drop. As detailed in the ablation analysis (Section 4.4, Tables 3–4), the aggregate gain is concentrated in a single category (Black rot of tea: mAP@50 0.878 → 0.995), while the remaining six categories already operate near saturation in the baseline and benefit primarily on the stricter mAP@50–95 metric. Inference latency on the i5-13500H CPU drops from 63.62 ms to 51.39 ms (−19.2%), supporting deployment feasibility on commodity edge hardware.

**Table 4:** Module-level ablation on the independent test set (1,547 images). Latency was measured on a 13th-Gen Intel Core i5-13500H CPU at 640 × 640 resolution, batch = 1, FP32, with no GPU acceleration or quantization.

Variant	CA	PD	Params (M)	GFLOPs	mAP@50 (%)	mAP@50–95 (%)	Precision	Recall	Latency (ms)	FPS
Baseline (YOLOv8n)	–	–	3.01	8.20	97.11	74.42	0.877	0.974	63.62	15.7
+ CA only	✓	–	3.02	8.21	98.74	72.40	0.971	0.839	63.69	15.7
+ PD only	–	✓	2.03	6.21	99.00	75.62	0.964	0.979	48.85	20.5
CA-YOLOv8n (Full)	✓	✓	2.03	6.22	98.89	76.26	0.946	0.986	51.39	19.5

#### 4.3.3 Analysis of computational efficiency.

To verify the potential for edge-device deployment, we compared the computational complexity and inference speed. As shown in Table 3 (with full per-module attribution in Table 4, Section 4.4), the proposed model achieves higher precision with substantially fewer parameters and FLOPs. All inference-speed tests were conducted on the same hardware platform (13th-Gen Intel Core i5-13500H, CPU only, 640 × 640 input, batch = 1, FP32).

GFLOPs measured at 640 × 640 inference resolution using ultralytics.utils.torch_utils.get_flops. The values previously reported (4.1 / 3.1) corresponded to a different input scale and have been corrected here for consistency with the inference pipeline. PPR = mAP@50 (%) / Parameters (M). Higher is better.

We contextualize CA-YOLOv8n against two representative works in the recent lightweight plant-disease literature. Xue et al. [28] proposed YOLO-Tea, a YOLOv5s-based detector incorporating ACmix, CBAM, RFB, and GCNet modules; on a two-class tea-disease dataset (tea leaf blight and green mirid bug, 450 images), YOLO-Tea reported mAP@50 = 79.3% with approximately 7.96 M parameters and a 15.6 MB model footprint. Guan et al. [19] proposed Dise-Efficient, a lightweight EfficientNetV2-derived network for plant disease and pest classification (not localization); on the 38-class Plant Village dataset, Dise-Efficient reported a top-1 accuracy of 99.80% at a 13.3 MB model size. We emphasize that these figures are drawn from each work's own dataset and that Dise-Efficient solves a classification rather than detection problem; they therefore serve as approximate positional context rather than direct comparison. Against this backdrop, CA-YOLOv8n attains 98.89% mAP@50 across eight tea-disease categories at 2.03 M parameters, 6.22 GFLOPs, and 51.39 ms CPU latency—an object-detection result on a richer class taxonomy and at a substantially smaller parameter footprint than YOLO-Tea, indicating a competitive operating point on the parameter-efficiency frontier of lightweight tea-disease analysis.

Despite achieving a comparable mAP@50 to YOLOv10n, CA-YOLOv8n delivers a more favorable balance of detection performance and model compactness, utilizing 74.8% fewer parameters and 49.8% less computational load (GFLOPs) while maintaining a substantially higher Recall (0.986 vs. 0.819). Notably, the gap in deployment-relevant inference latency is even more pronounced: CA-YOLOv8n attains a 3.48 × speedup over YOLOv10n on the same CPU platform (51.39 ms vs. 179.00 ms), confirming that the streamlined neck not only reduces theoretical FLOPs but also translates to substantially faster real-world inference on commodity edge hardware.

### 4.4 Ablation study

**Fig 10:**
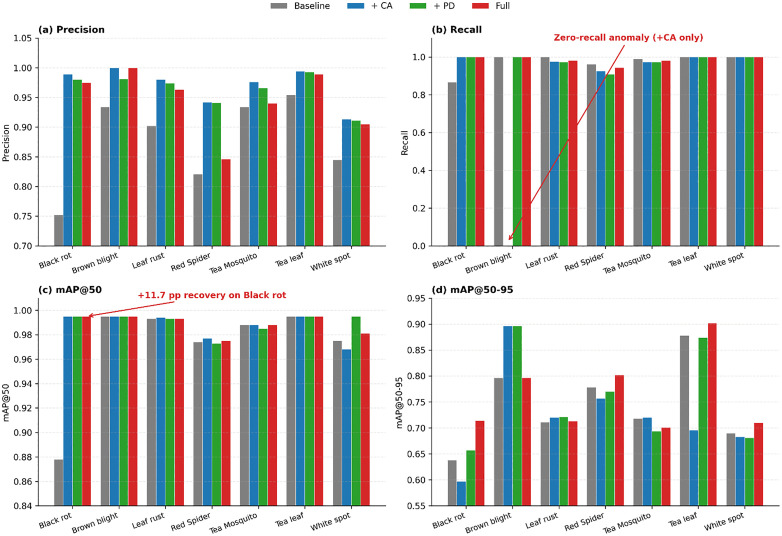
Per-class diagnostic performance of the four ablation variants on the independent test set. **(a)** Precision; **(b)** operating-point Recall, where the + CA-only variant exhibits a zero-Recall anomaly on the rarest category (*Brown blight of tea*) that is fully resolved by the addition of Path-Decoupling; **(c)** mAP@50, where the Baseline's failure on *Black rot of tea* (0.878) is fully recovered by all three modified variants (≥ 0.995); **(d)** mAP@50-95. The “disease” residual aggregate class contains no samples in the held-out test split and is therefore omitted.

To rigorously isolate the contribution of each architectural modification, we conducted a controlled 2 × 2 ablation in which the Coordinate Attention (CA) module and the Path-Decoupling (PD) strategy were independently enabled and disabled. All four variants—Baseline (vanilla YOLOv8n), + CA only, + PD only, and CA-YOLOv8n (the full model)—were trained from the same YOLOv8n pretrained weights, under identical hyperparameters, with a fixed random seed and deterministic mode enabled, on the same Kaggle dual-T4 platform. Aggregate results are reported in Table 4; per-class metrics are presented in Fig 10.

The four-way comparison reveals that CA and PD address two complementary failure modes of the baseline architecture, and that their combination delivers the most balanced operating point.

(i) Path-Decoupling alone delivers efficiency without sacrificing accuracy. Replacing the standard PAN-FPN neck with the Streamlined-Neck reduces parameters by 32.6% (3.01  →  2.03 M), GFLOPs by 24.3% (8.20  →  6.21), and CPU inference latency by 23.2% (63.62  →  48.85 ms), while simultaneously improving mAP@50 from 97.11% to 99.00% (+1.89 pp) and mAP@50-–95 from 74.42% to 75.62% (+1.20 pp). This counter-intuitive result—that removing fusion layers improves accuracy—indicates that the redundant feature aggregation in the original neck was diluting fine-grained lesion features rather than enriching them.
(ii) Coordinate Attention alone produces a mixed effect. Adding CA to the baseline lifts mAP@50 by 1.63 pp (97.11  →  98.74%) and average per-class Precision by 9.4 pp (0.877  →  0.971), without measurable change in parameter count or CPU latency. However, the same variant exhibits a 2.02 pp drop in mAP@50-–95 (74.42  →  72.40%) and a sharp drop in operating-point Recall (0.974  →  0.839)—the latter concentrated almost entirely in a single category, Brown blight of tea, where the maximum-F1 confidence threshold collapses to a regime that yields zero Recall (Fig 10). Because the area under the precision-recall curve for this category remains 0.995, the underlying class representation is intact; what changes is the placement of the optimal operating threshold. We interpret this as the cost of injecting strong directional encoding into a network whose neck has not been adapted to consume it: the CA-recalibrated features compete with the multi-scale aggregation in the original PAN-FPN, producing a bimodal confidence distribution for the rarest category. This observation is the empirical basis for our design choice to couple CA with the streamlined neck rather than to deploy it as a standalone enhancement.
(iii) The full CA-YOLOv8n combines both improvements without inheriting either weakness. Pairing CA with PD fully resolves the working-point instability observed in CA-only (Brown blight Recall returns to 1.000) and preserves the efficiency gains of PD-only. The full model achieves the highest Recall (0.986) and the highest mAP@50-–95 (76.26%) of any variant, while matching PD-only on parameter count and GFLOPs to within rounding. The slight Precision drop relative to PD-only (0.946 vs. 0.964) is the conventional consequence of prioritizing Recall, and is the operationally desirable trade-off for early-stage disease screening, where false negatives carry higher epidemiological cost than false positives. The 2.5 ms latency gap between PD-only and the full model (48.85 vs. 51.39 ms) lies within the run-to-run variance typical of single-thread CPU inference and does not reflect a structural cost of the CA module, which adds only 0.01 GFLOPs.

Per-category observations. Two patterns emerge from Fig 10 First, the 1.78 pp aggregate gain in mAP@50 between Baseline (97.11%) and the full model (98.89%) is dominated almost entirely by a single category—Black rot of tea—whose mAP@50 rises from 0.878 to 0.995 (+11.7 pp). For the remaining six categories the baseline already operates near saturation (mAP@50 ≥ 0.974), and the gains of the full model concentrate on the stricter mAP@50–95 metric, reflecting tighter bounding-box regression rather than coarser detection. This pattern is consistent with the design role of CA as a positional refinement mechanism that sharpens the localization of categories that were already detectable. Second, the Brown blight zero-Recall anomaly is exclusive to the + CA-only variant; both PD-only and the full model recover R = 1.000 on this category, confirming that PD's structural simplification is functionally necessary for CA's spatial encoding to translate into stable downstream detection.

Remark on the residual disease category. The dataset, as released by its public source, defines an eighth class labelled disease as a residual aggregate of pathologies that do not belong to the seven specific categories. Because no instances of this aggregate class appear in the held-out test split provided by the original distribution, per-class metrics in Fig 10 are reported for the seven semantically defined categories only. The class is retained in the training pipeline to preserve fidelity to the public-dataset specification, but its limited diagnostic informativeness is acknowledged in the limitations discussion.

### 4.5 System implementation and application demonstration

**Fig 11:**
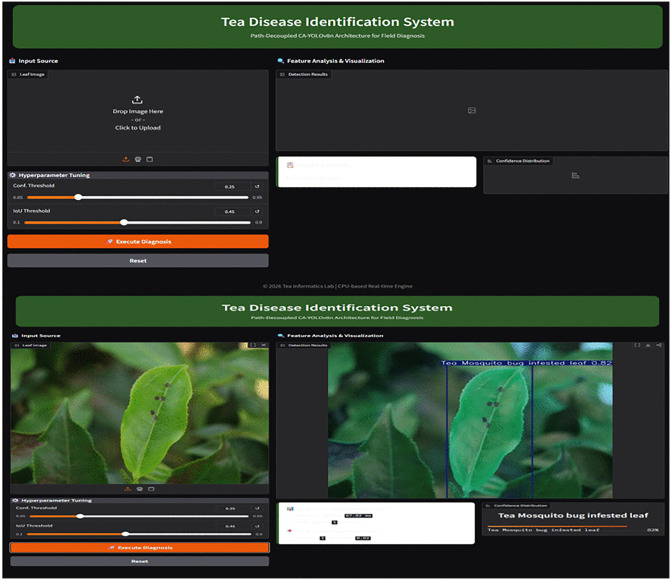
The GUI of the Tea Disease Identification System.

As illustrated in Fig 11, the system interface exposes the controls required for field use. The Input Source panel accepts leaf images for inference, and the Hyperparameter Tuning module provides dynamic adjustment of the confidence and IoU thresholds, allowing the model to be re-tuned for different field conditions (e.g., varying lighting or lesion density) without retraining.

#### 4.5.1 Intelligent diagnostic reporting.

A key feature of the system is the Diagnostic Intelligence Report, which provides localized feature anchoring and quantitative analysis.

Confidence Distribution: Unlike traditional counting methods, the system visualizes the Average Confidence Score for each pathology, providing a scientifically rigorous basis for diagnosis.

Professional Strategies: For each identified lesion, the system automatically correlates with a pathology knowledge base to provide specific management strategies (e.g., pruning or chemical control), transforming raw detection data into actionable agricultural decisions.

## 5. Conclusion and future work

### 5.1 Conclusion

We presented CA-YOLOv8n, a lightweight tea-leaf disease detector that combines Coordinate Attention as a Spatial-Aware attention block with a Path-Decoupling Streamlined-Neck on the YOLOv8n backbone. Trained and evaluated on the publicly hosted 9,591-image tea-leaf disease dataset across eight categories, the model produces two principal results.

1. Architectural redesign. Removing the full PAN-FPN neck of YOLOv8n and replacing it with a single Coordinate Attention block on the deepest backbone feature (P5) reduces parameters from 3.01 M to 2.03 M (–32.6%) and GFLOPs from 8.20 to 6.22 (–24.1%), while improving mAP@50 from 97.11% to 98.89% (+1.78 pp) and mAP@50–95 from 74.42% to 76.26% (+1.84 pp).
2. Complementary modular effects. The four-variant controlled ablation shows that Coordinate Attention raises Precision but exhibits a confidence-threshold sensitivity that drives Recall on the rarest category (Brown blight of tea) to zero, while Path-Decoupling lowers parameter count and latency without that Recall instability. The combined CA-YOLOv8n recovers the Precision gain from CA while preserving the Recall stability and the lightweight footprint of PD.

### 5.2 Limitations

Three limitations warrant explicit acknowledgement.

First, the dataset is drawn from a single public repository; cross-source generalisation to images captured under different illumination, camera sensors, or geographical and seasonal conditions has not been independently verified and should not be assumed.

Second, the residual disease aggregate class is semantically coarse and contains no instances in the held-out test split (a 4 / 0 / 0 train / valid / test split); no diagnostic claim is made about predictions of this label, and we treat it as a placeholder pending dataset refinement.

Third, the + CA-only ablation variant exhibits a confidence-threshold sensitivity on Brown blight of tea: its maximum-F1 operating point collapses to zero Recall. The instability is fully resolved when CA is paired with Path-Decoupling in the full model, but indicates that CA-style positional encoding cannot be deployed as a drop-in addition to a fixed PAN-FPN baseline.

### 5.3 Future work

Three directions remain. First, cross-dataset evaluation, repeated-seed training, formal cross-validation, and head-to-head retraining of YOLO-Tea and Dise-Efficient on the figshare-deposited dataset under an identical protocol, to strengthen the generalisation claim. Second, INT8 quantisation and ARM-CPU benchmarking on edge devices beyond the desktop-class CPU evaluated here, to extend the deployment envelope. Third, integration of a multimodal large language model that produces actionable pest-control advice conditioned on the per-class detection result.
