# Supplementary material for: Spatial-aware lightweight network for real-time tea disease detection: A coordinate attention-enhanced YOLOv8n approach with path-decoupling strategy
Source: PLoS One. 2026 Jul 24;21(7):e0354583. doi: 10.1371/journal.pone.0354583 (PMC13399476; doi:10.1371/journal.pone.0354583)
Supplement: Attachment — Submitted filename: RESPONSE_TO_REVIEWERS.docx [file pone.0354583.s002.docx]

# Response to Reviewers

**Manuscript:** PONE-D-26-12938 **Title:** Spatial-Aware Lightweight Network for Real-time Tea Disease Detection: A Coordinate Attention-Enhanced YOLOv8n Approach with Path-Decoupling Strategy **Authors:** Xiang Lyu, ChengLei Song, Yue Yu

Dear Dr. Xiaowei Li and the two anonymous Reviewers,

We thank the academic editor and both reviewers for the detailed, constructive comments on our submission. Each point has been addressed in the revised manuscript, and a marked-up copy with track changes has been uploaded alongside this letter. Where a comment is cross-referenced to a section, figure, or table, the section/figure/table number refers to the revised manuscript. Two new figures have been added during the revision — Fig 2 (the Path-Decoupling neck comparison requested by Reviewer #1, R1.5) and Fig 10 (the per-class diagnostic produced for the new ablation study, requested by Reviewer #1, R1.2). A new literature-summary **Table 1** has also been added at the end of Section 2 (in response to Reviewer #1, R1.1), so the three pre-existing tables have been renumbered: Comparative performance → Table 2, Computational efficiency → Table 3, Module-level ablation → Table 4. All previously existing figures from old Fig 2 onward have been renumbered upward by one (old Fig 2 → new Fig 3, etc.).

The point-by-point responses are organised below in three sections: 1. Academic editor’s journal requirements (items 1–7) 2. Reviewer #1 (seven comments) 3. Reviewer #2 (comments grouped by section)

A summary list of every change made to the manuscript is provided at the end of this letter.

## 1. Academic Editor — Journal Requirements

### Requirement 1. PLOS ONE style template

*“Please ensure that your manuscript meets PLOS ONE’s style requirements, including those for file naming.”*

**Authors’ response:** The manuscript has been reformatted to follow the PLOS ONE style template (main body and title/authors/affiliations samples linked in the decision letter). Section headings, paragraph spacing, figure/table captions, and reference formatting now conform to the PLOS ONE guidelines.

### Requirement 2. Author-generated code must be openly available

*“Please note that PLOS One has specific guidelines on code sharing for submissions in which author-generated code underpins the findings in the manuscript. We expect all author-generated code to be made available without restrictions upon publication of the work.”*

**Authors’ response:** A reference implementation has been released publicly on GitHub under the GNU Affero General Public License v3.0 (the licence required by the Ultralytics YOLOv8 framework on which the work builds):

**Repository:** <https://github.com/lvxiangfly/tea-disease-detection> **Release tag:** v1.0.0 **Direct link to the release snapshot:** <https://github.com/lvxiangfly/tea-disease-detection/releases/tag/v1.0.0>

The release contains the Coordinate-Attention module, the CA-YOLOv8n model YAML, the original Kaggle training notebook that produced the checkpoint reported in this paper, portable training/evaluation/inference scripts, the CPU latency benchmark script used for Table 2, and the final trained weight file.

### Requirement 3. Dataset must be deposited in a PLOS-acceptable repository

*“Unfortunately, the repository you have noted in your Data Availability statement does not qualify as an acceptable data repository according to PLOS’s standards. At this time, please upload the minimal data set necessary to replicate your study’s findings to a stable, public repository (such as figshare or Dryad).”*

**Authors’ response:** The complete 9,591-image tea-leaf disease dataset has been deposited on figshare under DOI:

**DOI:** 10.6084/m9.figshare.32253357 **URL:** <https://doi.org/10.6084/m9.figshare.32253357> **Licence:** CC BY 4.0

### Requirement 4. ORCID for corresponding author validated in Editorial Manager

*“PLOS requires an ORCID iD for the corresponding author in Editorial Manager on papers submitted after December 6th, 2016.”*

**Authors’ response:** An ORCID iD for the corresponding author has been registered at orcid.org and will be validated via the Fetch/Validate link in Editorial Manager prior to resubmission.

### Requirement 5. Figure copyright check (Figures 2, 3, 6, 8, 9 in the original submission)

*“We note that Figures 2, 3, 6, 8, and 9 in your submission contain images which may be copyrighted… We require you to either (1) present written permission from the copyright holder to publish these figures specifically under the CC BY 4.0 license, or (2) remove the figures from your submission.”*

**Authors’ response:** After re-examining all flagged figures, we clarify the situation as follows. The five figures cited in the editor’s note (using the original submission’s numbering) correspond to:

| Original submission # | Revised manuscript # | Caption (short) | Authorship |
| --- | --- | --- | --- |
| Fig 2 | **Fig 3** | Architectural framework of the tea-disease identification system | **Original — drawn by the authors** |
| Fig 3 | **Fig 4** | Detailed inference pipeline and dynamic threshold decision-making logic | **Original — drawn by the authors** |
| Fig 6 | **Fig 7** | Batch Samples | Composed of dataset images (see below) |
| Fig 8 | **Fig 9** | Qualitative comparison of detection results | Composed of dataset images (see below) |
| Fig 9 | **Fig 11** | The GUI of the Tea Disease Identification System | **Original — author’s software screenshot** |

Originally created by the authors (no third-party content reused). Figs 1,2, 3, 4, 5,6,8, 10, and 11 of the revised manuscript — comprising the architectural diagrams, the confusion matrix, the per-class diagnostic, and the GUI screenshot — were produced entirely by the authors using draw.io, matplotlib, and our own GUI application, so the CC BY 4.0 licence applies without restriction.

Drawn from the authors' figshare dataset. Figs 7 and 9 incorporate sample images from the tea-leaf disease dataset deposited at figshare DOI 10.6084/m9.figshare.32253357 (CC BY 4.0). Per the editorial office's latest request prior to peer review, we have now explicitly added the relevant attribution, DOI, and usage information (CC BY 4.0) directly into the captions of Fig 7 and Fig 9 in the revised manuscript.

### Requirement 6. Table 2 must be referred to in the text

*“We note you have included a table to which you do not refer in the text of your manuscript. Please ensure that you refer to Table 2 in your text.”*

**Authors’ response:** The table the editor flagged (Analysis of computational efficiency and parameter utilization) — originally Table 2, now **Table 3** in the revised manuscript after a new literature-summary Table 1 was added at the end of Section 2 — is now explicitly cited in **Section 4.3.2 (Analysis of Computational Efficiency)**, which discusses each column of the table and links the parameter-efficiency ratio (PPR) of CA-YOLOv8n to the Path-Decoupling design.

### Requirement 7. Missing Table 4.5 referenced on page 15

*“Please include a copy of Table 4.5 which you refer to in your text on page 15.”*

**Authors’ response:** The original reference to “Table 4.5” was a typographical placeholder that was no longer needed once the ablation analysis was consolidated. The reference has been removed, and the ablation results are now presented in **Table 4 (Module-level ablation on the independent test set)** of the revised manuscript and complemented by **Fig 10 (Per-class diagnostic performance of the four ablation variants)**. No further “Table 4.5” reference appears anywhere in the revised text.

## 2. Reviewer #1

### R1.1. Additional literature and a literature-review summary table

*“The research is interesting; however, it would benefit from the inclusion of more relevant literature to ensure that the context is comprehensively covered. It is also recommended to include a summary table at the end of the literature review to compare existing approaches, which would help to clearly position the proposed method within the current body of research.”*

**Authors’ response:** The Related Work section has been expanded along three lines. (i) Additional references covering attention-based pest/disease detection, lightweight YOLO variants for agricultural deployment, and tea-domain works have been added. (ii) A new comparative-summary **Table 1 (Comparison of existing models for agricultural disease detection)** has been inserted at the end of Section 2; it compares five representative recent models across four axes (base architecture, key enhancements, and limitations) — *YOLO-Tea* (Xue et al., 2023) , *YOLOv8-RCAA* (Wang et al., 2024) , *MobileNet-CA-YOLO* (Jia et al., 2023) , *Dise-Efficient* (Guan et al., 2023) , and *AX-RetinaNet* (Bao et al., 2022) — three of which (YOLO-Tea, YOLOv8-RCAA, AX-RetinaNet) are explicitly tea-disease detectors, providing direct in-domain positioning. (iii) The narrative in Section 2 now references each row of Table 1 and articulates how the proposed CA-YOLOv8n is positioned relative to these works (notably, Coordinate Attention is *coupled with neck-side Path-Decoupling* rather than inserted into the backbone as in MobileNet-CA-YOLO). Because Table 1 has been inserted at the start of the numbered table sequence, the three pre-existing tables have been renumbered accordingly (Comparative performance → **Table 2**; Computational efficiency → **Table 3**; Module-level ablation → **Table 4**), and all in-text and Response-letter references below use the revised numbering.

### R1.2. Ablation study isolating each module

*“The paper introduces multiple enhancements (coordinate attention, path-decoupling, lightweight design), but it is not entirely clear how each component individually contributes to performance. Consider adding an ablation study to isolate the impact of each module.”*

**Authors’ response:** A controlled 2 × 2 ablation has been added in **Section 4.4 (Ablation Study)** of the revised manuscript. The four variants — *Baseline (YOLOv8n)*, *+ CA only*, *+ PD only*, and *CA-YOLOv8n (Full)* — share an identical training protocol and are reported together in **Table 4** (parameters, GFLOPs, mAP@50, mAP@50-95, Precision, Recall, Latency, FPS) and in **Fig 10** (per-class Precision / Recall / mAP@50 / mAP@50-95). The ablation isolates the contributions of each component: the +CA-only variant raises mAP@50 from 97.11 % to 98.74 % while leaving the parameter count essentially unchanged (3.01 M → 3.02 M); the +PD-only variant reduces parameters and GFLOPs by 33 % and 24 % respectively (3.01 M → 2.03 M, 8.20 → 6.21 GFLOPs) and cuts CPU latency from 63.62 ms to 48.85 ms; combining the two recovers the precision gain while preserving the lightweight footprint of PD (2.03 M / 6.22 GFLOPs / 51.39 ms / mAP@50 = 98.89 %, mAP@50-95 = 76.26 %). The complementary failure modes the two modules address (CA improves Precision, PD improves Recall) are discussed in Section 4.4.

### R1.3. Technical definition of “Spatial-Aware”

*“The term ‘spatial-aware’ is used prominently in the title, but its technical definition and implementation are not sufficiently explained. Clarify how spatial awareness is modeled differently from standard YOLOv8 attention mechanisms.”*

**Authors’ response:** Two paragraphs have been added at the opening of **Section 3.3 (Coordinate Attention and Architectural Compensation)** to define the term and contrast it with the channel-only attention typically paired with YOLOv8. Briefly: standard channel-attention modules such as Squeeze-and-Excitation (Hu et al., 2018) collapse every spatial location of a channel into a single scalar via global average pooling, so the resulting gating signal is **position-agnostic** within a channel. Coordinate Attention replaces that 2D global pooling with two complementary 1D pooling kernels of size (H, 1) and (1, W), which aggregate features along the vertical and horizontal axes separately; the direction-aware descriptors are concatenated, passed through a shared 1 × 1 convolution, and split back into a height-wise and a width-wise attention map, each broadcast over its orthogonal axis when modulating the input. The recalibration weights therefore vary across spatial coordinates rather than being shared within a channel — this row- and column-conditioned gating is the precise sense in which the proposed network is *Spatial-Aware*, and it is what distinguishes Coordinate Attention from SE and from the position-agnostic attention designs commonly inserted into YOLOv8. Section 3.3 then formalises the two stages of CA (Coordinate Information Embedding in Section 3.3.1 and Coordinate Attention Generation in Section 3.3.2) before integrating its output under the Path-Decoupling design illustrated in Fig 2.

### R1.4. Justification for choosing YOLOv8n (nano) over s / m variants

*“The choice of enhancing YOLOv8n specifically should be better justified. Why is the nano version preferred over other variants (e.g., YOLOv8s/m)? Is the focus primarily on edge deployment or computational constraints?”*

**Authors’ response:** The choice is dictated by the target deployment scenario stated in the Introduction (Section 1) and reinforced in Section 4.3 (Performance Comparison): a CPU-only edge platform represented in this study by an Intel Core i5-13500H. The nano variant is the only scale of the YOLOv8 family whose parameter footprint (~3 M) and FLOP budget (~8 GFLOPs) keep single-image CPU latency within the sub-100 ms range that the agricultural inspection workflow targeted in this paper requires. YOLOv8s (~11 M parameters, ~28 GFLOPs) and YOLOv8m (~26 M, ~79 GFLOPs) would multiply CPU latency by roughly 4 × and 12 × respectively.

### R1.5. Diagram / architectural comparison of the Path-Decoupling strategy

*“The proposed path-decoupling strategy needs clearer explanation. A diagram or architectural comparison would help readers understand how it differs from conventional detection heads.”*

**Authors’ response:** A side-by-side architectural comparison has been added as **Fig 2** in the revised manuscript. Panel (a) depicts the standard PAN-FPN neck used by YOLOv8n — 12 successive operations (2 Upsample + 4 Concat + 4 C2f + 2 Conv-down) sitting between the backbone outputs (P3, P4, P5) and the three detection heads. Panel (b) depicts the proposed Streamlined-Neck: the entire PAN-FPN neck is removed, P3 (layer 4) and P4 (layer 6) are routed directly from the backbone into the detection heads, and only a single Coordinate-Attention block is inserted on the deepest backbone feature P5. The figure caption quantifies the cost differential (≈ 0.98 M parameters and ≈ 1.98 GFLOPs removed; 12 fusion layers replaced by 1 attention layer), and the Path-Decoupling rationale is now formalised in **Section 3.3.3 (Feature Recalibration under Path-Decoupling)**. The matplotlib script used to render Fig 2 is included in the public code release (docs/architecture.png / .pdf) for full reproducibility.

### R1.6. Edge deployment feasibility, real-world conditions, and Limitations

*“Since the work targets real-time tea disease detection, discuss: Deployment feasibility on edge devices; Performance under real-world conditions (e.g., varying weather, leaf overlap). Explain further on this. Include a brief discussion on limitations of the study.”*

**Authors’ response:** All three sub-points have been addressed in the revised manuscript.

- **Edge deployment feasibility.** The subsection *Performance Advantages of Lightweight Deployment* (now part of the System Implementation and Deployment block in Section 3) lists the exact deployment platform (Intel Core i5-13500H CPU, single thread, batch = 1, FP32, 640 × 640) and reports the achieved end-to-end inference latency (51.39 ms ≈ 19.5 FPS). Section 4.3 (Performance Comparison) further benchmarks CA-YOLOv8n against YOLOv8n and YOLOv10n on the same hardware (51.39 ms vs. 63.62 ms and 179.00 ms; see Table 2), confirming that the proposed design meets the sub-100 ms real-time budget on commodity CPU hardware without GPU acceleration.
- **Performance under real-world conditions.** **Fig 9** (Qualitative comparison of detection results between baseline YOLOv8n and the proposed CA-YOLOv8n under complex tea plantation environments) presents detection examples that include occluded leaves, overlapping foliage, and varying illumination. The accompanying narrative in Section 4.3.1 walks through several representative cases where the baseline either misses heavily overlapping lesions or misclassifies them, and shows the corresponding CA-YOLOv8n predictions. The class-imbalance discussion newly added to Section 3.1.1 (Statistical Distribution) and the confusion-matrix analysis in Section 4.2 further quantify the failure modes that remain under real-world distribution shift.
- **Limitations.** A dedicated **Section 5.2 (Limitations)** has been added to the revised manuscript. It explicitly acknowledges (i) reliance on a single public dataset, with cross-source generalisation not independently verified; (ii) the residual *disease* aggregate class, which contains no instances in the held-out test split (a 4 / 0 / 0 train / valid / test split) and is therefore retained only as a placeholder; and (iii) a confidence-threshold sensitivity exhibited by the +CA-only ablation variant on the rarest category (*Brown blight of tea*), which collapses Recall to zero in that variant but is fully resolved in the full CA-YOLOv8n when CA is paired with Path-Decoupling.

### R1.7. Multiple experimental setups for robustness

*“Therefore, it is recommended to conduct multiple experimental setups to further validate the robustness of the proposed method and potentially improve the overall results. This could include varying training configurations, testing under different conditions, or evaluating on additional datasets.”*

**Authors’ response:** Two complementary sources of comparative evidence on the same dataset are reported in the revised manuscript: (i) the **four-variant controlled ablation** under an identical training protocol — *Baseline (YOLOv8n)*, *+ CA only*, *+ PD only*, *CA-YOLOv8n (Full)* — quantified in **Table 4** and **Fig 10**, which isolates the marginal contribution of each architectural component while keeping the training configuration fixed; and (ii) the **three-model head-to-head comparison** (YOLOv8n / YOLOv10n / CA-YOLOv8n) on the held-out 1,547-image test split, reported in **Tables 2 and 3** of Section 4.3 (Performance Comparison). Qualitative robustness under occlusion, overlapping foliage, and varying illumination is illustrated in **Fig 9**. We agree with the reviewer that the present evidence does not yet include cross-dataset evaluation, repeated-seed training, or formal cross-validation, and we have made this gap explicit in **Section 5.2 (Limitations)** and named it as the immediate next step in **Section 5.3**

## 3. Reviewer #2

Reviewer #2’s comments are grouped into seven thematic blocks (A–G), following the structure of the original review. Each comment is quoted verbatim and addressed individually below.

### Block A — Dataset (Section 3.1)

#### R2.A.1. Empty subsection “3.1.1 Statistical Distribution”

*“No statistical distribution figure is present despite ‘3.1.1 Statistical Distribution’ being listed as a subsection header with no content. This is a placeholder left unfilled.”*

**Authors’ response:** Section 3.1.1 (Statistical Distribution) has been filled in. It now provides a per-split, per-class instance breakdown across all eight categories, ranging from *Tea Mosquito bug* (7,254 instances, the dominant class) down to *Brown blight* (8 instances; only 4 in the training split) and the residual *disease* aggregate (4 instances in the training split, none in valid/test). The accompanying narrative makes the class imbalance explicit and identifies the small-support classes that re-appear in the confusion-matrix analysis of Section 4.2.

#### R2.A.2. Missing class-distribution / class-imbalance discussion

*“Class distribution of the dataset is not discussed, potentially ignoring any class imbalance issues. Eight classes across 9,591 images with no per-class breakdown is a significant omission.”*

**Authors’ response:** A dedicated paragraph on class imbalance has been added at the end of Section 3.1.1. The newly inserted per-split, per-class table makes the skew explicit: *Tea Mosquito bug* is the dominant class by a wide margin (5,501 / 585 / 1,168 train/valid/test instances), whereas *Brown blight* (4 / 0 / 4) and the residual *disease* aggregate (4 / 0 / 0) are extreme minority classes whose training counts fall below any threshold at which reliable per-class learning could be expected. The new text acknowledges this imbalance as a confounding factor in the per-class metrics, motivates the use of mAP@50 as the primary aggregate metric, and forward-links to the failure-mode analysis in Section 4.2 —where the zero-TP behaviour on the disease class, the elevated false-negative rates on the small-support Brown blight class, and the false-positive cost incurred on background regions by the dominant Tea Mosquito bug / Red Spider classes are examined directly (see also Response R2.D.1 and R2.D.2 below)

#### R2.A.3. Dataset has no DOI or verifiable link

*“The dataset is sourced from ‘Tencent Developer Community’ with no DOI, or verifiable link. Reproducibility is impossible without this.”*

**Authors’ response:** The complete 9,591-image dataset — together with the YOLO-format labels, the train/valid/test split folders, and the data.yaml descriptor required to reproduce every experiment in the manuscript — has been deposited on figshare under DOI **10.6084/m9.figshare.32253357** (CC BY 4.0; <https://doi.org/10.6084/m9.figshare.32253357>). The Data Availability Statement in the submission system has been updated accordingly.

### Block B — Experimental Design (Section 3)

#### R2.B.1. Confidence-threshold ablation and the missing Section 4.5

*“The confidence threshold of ≥0.25 is presented as a design choice, but its effect on precision is not rigorously quantified in a dedicated ablation — it is only briefly discussed in Section 3.6.2. Although the authors referred to Section 4.5 for relevant analysis, but the article do not contain any such section.”*

**Authors’ response:** Two distinct points are addressed here.

- *Orphan forward reference to “Section 4.5”.* The reference was a typographical residue from an earlier draft and has been removed. The threshold rationale is now consolidated in **Section 3.7.2 (Inference Logic and Model Integration)**.
- *Effect of the 0.25 threshold on Precision.* The primary aggregate metrics reported in Section 4.3 — **mAP@50** and **mAP@50-95** — integrate over the full confidence-score range, so they are independent of the 0.25 operating point; the relative ranking between Baseline, +CA, +PD, and CA-YOLOv8n (Full) in Tables 2 and 4 is therefore invariant to threshold choice. The 0.25 value is retained only for the deployment-time decision logic in the GUI (where a single hard threshold is required), following the Ultralytics default. A dedicated threshold sweep would refine the deployment-time analysis without altering the headline mAP results, and we have flagged it as an engineering refinement in **Section 5.3 (Future Work)**.

#### R2.B.2. Training hardware not specified

*“No training hardware specification is provided (only inference hardware in Section 4.3.3). Training reproducibility is compromised.”*

**Authors’ response:** Section 3.5 (Training Setup and Hardware Environment) has been rewritten to include the full training configuration: a Kaggle dual-GPU T4 instance running CUDA 12 with PyTorch and the Ultralytics YOLOv8 framework; 150 epochs with early-stopping patience = 30; image size 640 × 640; mosaic augmentation closed during the final 10 epochs; AMP disabled. The complete args.yaml and the verbatim Kaggle training notebook (notebooks/train_kaggle.ipynb) are included in the public code release at <https://github.com/lvxiangfly/tea-disease-detection> (release tag v1.0.0), so the exact training run that produced the shipped checkpoint can be reproduced or audited.

#### R2.B.3. No cross-validation or repeated-trial protocol

*“No cross-validation or repeated trial protocol is described. Results from a single train/test split are insufficient to establish generalizability claims.”*

**Authors’ response:** We agree, and the gap has been made explicit in the revised manuscript. Section 5.2 (Limitations) now states that the present evaluation uses a single train/valid/test split and a single random seed per ablation variant; Section 5.3 (Future Work) names cross-dataset evaluation, repeated-seed training, and formal cross-validation as the immediate next-step work. The same point is addressed in greater detail in our Response to Reviewer #1, Comment R1.7.

### Block C — Comparative Analysis (Section 4.3)

#### R2.C.1. Table 1 too narrow — missing comparison with YOLO-Tea / Dise-Efficient

*“Table 1 compares only three models (YOLOv8n, YOLOv10n, CA-YOLOv8n). No comparison against other lightweight tea-specific models (e.g., YOLO-Tea from reference [28], Dise-Efficient from [19]) is made, despite these being directly cited in the related work.”*

**Authors’ response:** The two tea-specific baselines mentioned by the reviewer have been incorporated into **Section 2 (Related Work)** as architectural / design-philosophy comparisons against CA-YOLOv8n, and both now appear as rows of the newly added literature-summary **Table 1** (see also Response R1.1). They were intentionally not added as new rows of the comparative-performance table (**Table 2** in the revised manuscript) because YOLO-Tea (Xue et al., 2023) and Dise-Efficient (Guan et al., 2023) were originally trained and evaluated on different tea-disease datasets with different category definitions. We have therefore provided the qualitative / architectural comparison in Section 2 and Table 1.

#### R2.C.2. Inconsistency between the 18.09 % improvement claim and Table 1

*“The 18.09% mAP improvement cited in Section 3.5.1 does not match the calculated difference from Table 1 (83.7% → 98.9% = 15.2 percentage points). Please explain the inconsistency in the reporting.”*

**Authors’ response:** The reviewer is correct on both counts — the 18.09 % figure was erroneous, and the original Baseline number (83.7 %) was outdated. The Baseline (YOLOv8n) row of the previous Table 1 had been computed from an earlier training configuration that no longer matched the rest of the experimental protocol. In the revised manuscript, **the YOLOv8n baseline has been retrained under the identical training protocol used for the other ablation variants** (Kaggle T4 × 2, 150 epochs, patience = 30, imgsz = 640, AMP off, mosaic closed in the last 10 epochs), and now reports **mAP@50 = 97.11 %**, **mAP@50-95 = 74.42 %**, **Precision = 0.877**, **Recall = 0.974**, **Latency = 63.62 ms**. Under this corrected baseline, the mAP@50 improvement of CA-YOLOv8n is **+1.78 pp** (97.11 % → 98.89 %), accompanied by a **+1.84 pp** improvement on mAP@50-95 (74.42 % → 76.26 %) and an additional **−19.2 %** CPU latency (63.62 ms → 51.39 ms). The “18.09 %” wording has been removed from the manuscript; the only mAP-improvement figures cited in the revised text are the corrected +1.78 pp / +1.84 pp values, which appear consistently in Section 4.3, Section 4.4 (Ablation Study), Table 2, Table 4, and Fig 10. We thank the reviewer for catching this discrepancy — the entire comparative narrative has been rewritten around the corrected baseline so that every numerical claim is now traceable to the underlying tables.

#### R2.C.3. Inference speed (FPS / ms latency) should be central, not peripheral

*“When reporting computational efficiency, inference speed (FPS/ms latency), which is central to the edge deployment problem.”*

**Authors’ response:** Inference speed has been promoted to a primary axis of every comparative table:

- **Table 2** (head-to-head on the test split) now reports per-image **CPU Latency (ms)** and **FPS** for YOLOv8n, YOLOv10n, and CA-YOLOv8n alongside the accuracy metrics — measured on a single thread of the Intel Core i5-13500H CPU at batch = 1, FP32, 640 × 640.
- **Table 3** (computational efficiency and parameter utilisation) reports parameter count, GFLOPs, and the parameter-precision ratio (PPR).
- **Table 4** (ablation study) reports Latency and FPS for all four ablation variants (Baseline / +CA only / +PD only / Full).

The accompanying narrative in Section 4.3 quantifies the deployment-relevant gaps explicitly: CA-YOLOv8n achieves a **3.48 × CPU latency advantage** over YOLOv10n (51.39 ms vs. 179.00 ms) and a **−19.2 % latency reduction** over the YOLOv8n baseline (63.62 ms → 51.39 ms). The Path-Decoupling-only variant in Table 4 further isolates the share of this latency reduction attributable to the architectural simplification of the neck (48.85 ms; −23.2 %), distinguishing it from the additional cost introduced when the CA module is added on top (+2.54 ms in the Full variant).

### Block D — Confusion Matrix (Section 4.2)

#### R2.D.1. *disease* class shows no true positives

*“The ‘disease’ (other minor diseases) category shows no true positive values in the confusion matrix (Fig. 7) and should be discussed.”*

**Authors’ response:** The reviewer’s observation is correct and is now discussed explicitly in **Section 4.2 (Confusion Matrix and Classification Detailed Analysis)** of the revised manuscript. The *disease* category is a residual aggregate that absorbs minor pathologies not covered by the seven specific classes; the newly added **Section 3.1.1 (Statistical Distribution)** makes clear that this class contains only **4 training instances, 0 validation instances, and 0 test instances** (a 4 / 0 / 0 split). The “no true positive values” pattern in the confusion matrix (now **Fig 8** in the revised manuscript, after the insertion of the new Fig 2) is therefore a structural consequence of the class having no test-set ground truth to recover, rather than a learning failure of the proposed model. The new Fig 10 (Per-class diagnostic) explicitly omits the *disease* class for this reason — its caption states “the *disease* residual aggregate class contains no samples in the held-out test split and is therefore omitted”. Section 4.2 mirrors this statement, and **Section 5.2 (Limitations)** lists the *disease* class as a deliberately retained but under-represented category that would benefit either from substantially more samples or from being merged into one of the seven specific categories in a follow-up dataset revision. We retained the class in the present submission to preserve backward compatibility with the public dataset’s original class definitions, which are now accessible on figshare.

#### R2.D.2. High false-negative rates on Tea Mosquito bug (0.52) and Red Spider (0.27)

*“False negative rates for Tea Mosquito bug (0.52) and Red Spider (0.27) are high and warrant deeper analysis.”*

**Authors’ response:** The 0.52 and 0.27 values cited by the reviewer do appear in the revised manuscript’s **Fig 8** (normalised confusion matrix), but they sit in the *background column* and represent the model’s **false-positive distribution on true background regions** — not the False Negative rates of these classes. Under Ultralytics’ default column-normalisation, the diagonal True-Positive rates for *Tea Mosquito bug* and *Red Spider* are **0.98** and **0.94** respectively, and the corresponding entries in the *background row* (i.e., the actual per-class False Negative rates) are **0.02** and **0.06** — consistent with the aggregate Recall of 0.986 reported in Table 2. The class-imbalance bias of the training distribution does manifest as a false-positive cost on visually ambiguous background (52% of background regions are predicted as *Tea Mosquito bug*, 27% as *Red Spider*, mirroring the per-class instance counts in Section 3.1.1), and this trade-off is acknowledged in Section 5.2 (Limitations).

The full per-class breakdown across the four ablation variants is reported in **Fig 10 (Per-class diagnostic performance on the independent test set)**: panel (a) reports per-class Precision and panel (b) reports per-class operating-point Recall. Fig 10(b) further shows that the **+CA-only variant exhibits a zero-Recall anomaly on the rarest category (*Brown blight of tea*, 4 / 0 / 4 split)**, which is **fully resolved by the addition of Path-Decoupling** in both the +PD-only and Full variants — direct evidence that PD is the component responsible for Recall recovery on rare classes. **Fig 10(c)** additionally shows that the Baseline’s per-class failure on *Black rot of tea* (mAP@50 = 0.878) is fully recovered by all three modified variants (≥ 0.995). Section 4.2 attributes residual elevated-FN behaviour on Brown blight and the omitted disease class to the very small number of training/test instances available rather than to an architectural failure of CA-YOLOv8n.

### Block E — Efficiency Metrics

#### R2.E.1. IEI metric defined but never reported

*“IEI metric was defined but never actually reported in any table.”*

**Authors' response:** We agree this was an inconsistency. In the revised manuscript, the IEI definition has been removed from §3.6 and replaced by three concrete edge-deployment metrics — CPU Latency (ms), FPS, and the Parameter–Precision Ratio (PPR). Latency and FPS are now reported for every model in Tables 2, and 4, and PPR is reported in Table 3. Every metric defined in §3.6 has a corresponding value in the comparative tables of Section 4; no orphan definition remains.

### Block F — Deployment

#### R2.F.1. “Professional agricultural standards” — please specify

*“As mentioned, the system interface was developed following ‘professional agricultural standards’. Please specify which standards are you referring to.”*

**Authors’ response:** The reviewer is correct that the phrase “professional agricultural standards” was unsupported by a specific citation. The corresponding sentence in **Section 4.5 (System Implementation and Application Demonstration)** has been rewritten to describe the GUI’s controls directly without the qualitative claim: the Input Source panel accepts leaf images for inference, the Hyperparameter Tuning module provides dynamic adjustment of the confidence and IoU thresholds, and the GUI permits re-tuning for different field conditions (e.g., varying lighting or lesion density) without retraining the model. The complementary description in **Section 3.7.3 (Interactive Interface and Multi-mode Diagnostics)** also lists the four operational features of the interface (per-class confidence scores, per-image inference latency display, lesion bounding boxes overlaid on the input image, and a textual diagnostic report keyed to the dominant detected class).

#### R2.F.2. Hardware / software details of the edge platform

*“The authors mentioned about deployment on the edge, please provide more hardware/software details of the edge platform used.”*

**Authors’ response:** The edge platform is now fully specified in **Section 3.7.4 (Performance Advantages of Lightweight Deployment)** and **Section 4.3 (Performance Comparison)**:

- **Hardware.** Intel Core i5-13500H CPU (13th-generation, hybrid P-/E-core architecture). All latency measurements use a single thread at batch = 1, image size 640 × 640, FP32 precision; no GPU acceleration is used.
- **Software.** Python ≥ 3.10, PyTorch (CPU build), the Ultralytics YOLOv8 framework, and the public CA-YOLOv8n release at <https://github.com/lvxiangfly/tea-disease-detection> (release tag v1.0.0, AGPL-3.0). The GUI prototype shown in Fig 11 is built on the Gradio web-UI library.

Aggregate end-to-end inference latency on this platform is **51.39 ms / 19.5 FPS** for CA-YOLOv8n (Table 2). **Section 5.3 (Future Work)** notes INT8 quantisation and ARM-CPU benchmarking as planned engineering steps to extend the deployment envelope to smaller edge devices.

### Block G — Language

#### R2.G.1. Hyperbolic phrasing inappropriate for scientific publication

*“Writing is generally intelligible but contains hyperbolic phrasing inappropriate for scientific publication (e.g., ‘dramatic improvement,’ ‘impressive 98.9%,’ ‘a robust and efficient solution’).”*

**Authors’ response:** We agree. The three specific phrases highlighted by the reviewer have been revised in the manuscript, and a manuscript-wide pass has been performed to identify and correct other similar hyperbolic wording throughout the text.

## 4. Summary of Changes

The following is a consolidated list of every change made to the manuscript between the original submission and the revised version. References are to the section / figure / table numbering of the **revised** manuscript.

### 4.1 New figures

- **Fig 2** — *Architectural comparison between the standard PAN-FPN neck (YOLOv8n baseline) and the proposed Path-Decoupling Streamlined-Neck (CA-YOLOv8n).* Inserted in response to Reviewer #1, Comment R1.5. The matplotlib script that renders the figure (docs/architecture.png and .pdf) is included in the public code release for reproducibility.
- **Fig 10** — *Per-class diagnostic performance of the four ablation variants on the independent test set (Precision / Recall / mAP@50 / mAP@50-95).* Inserted in response to Reviewer #1, Comment R1.2, and complements Table 4 of the new ablation study.

### 4.2 New / rewritten sections

- **Section 2 (Related Work)** — expanded with additional references on attention-based pest/disease detection, lightweight YOLO variants, and tea-domain methods. A new comparative-summary **Table 1 (Comparison of existing models for agricultural disease detection)** has been inserted at the end of Section 2 in response to Reviewer #1, Comment R1.1. Three pre-existing tables have been renumbered: Comparative performance → Table 2; Computational efficiency → Table 3; Module-level ablation → Table 4.
- **Section 3.1.1 (Statistical Distribution)** — filled in with the per-split, per-class instance breakdown and the accompanying class-imbalance discussion.
- **Section 3.3 (Coordinate Attention and Architectural Compensation)** — two new opening paragraphs added to define *Spatial-Aware* mechanistically and contrast it with SE-style global attention.
- **Section 3.5 (Training Setup and Hardware Environment)** — rewritten to specify the full training configuration (Kaggle T4 × 2, 150 epochs, patience = 30, image size 640 × 640, AMP off, mosaic closed in the last 10 epochs).
- **Section 4.4 (Ablation Study)** — new section reporting the four-variant ablation (*Baseline* / *+ CA only* / *+ PD only* / *Full*).
- **Section 4.5 (System Implementation and Application Demonstration)** — the GUI description has been rewritten to remove the unsupported “professional agricultural standards” claim and to describe the interface controls concretely.
- **Section 5.2 (Limitations)** — new dedicated section listing the three current limitations (single public dataset / unverified cross-source generalisation, residual *disease* aggregate class, and a Recall collapse on the rarest category that appears only in the +CA-only ablation variant and is fully resolved in the full CA-YOLOv8n.
- **Section 5.3 (Future Work)** — expanded to commit to head-to-head retraining of YOLO-Tea and Dise-Efficient on the figshare-deposited dataset, cross-validation, repeated-seed training, INT8 quantisation, and ARM-CPU benchmarking.

### 4.3 Numerical revisions

- The YOLOv8n baseline has been retrained under the same training protocol as the other ablation variants. The Baseline row in Table 2, the per-class metrics in the confusion matrix (Fig 8), the per-class diagnostic in Fig 10, and the descriptive narrative in Section 4.3 have all been updated to the corrected values (mAP@50 = 97.11 %, Recall = 0.974, Latency = 63.62 ms).
- The erroneous “18.09 % mAP improvement” claim has been removed. The corrected mAP@50 improvement of CA-YOLOv8n over the retrained baseline is **+1.78 percentage points** (97.11 % → 98.89 %); the mAP@50-95 improvement is **+1.84 pp** (74.42 % → 76.26 %).
- **Table 2** (YOLOv10n row): the previously empty *Latency (ms)* and *FPS* cells have been measured under the unified inference pipeline and filled in (179.00 ms, 5.6 FPS).
- **Table 4** (new): reports the four-variant ablation with parameter count, GFLOPs, mAP@50, mAP@50-95, Precision, Recall, Latency, and FPS for each variant.

### 4.4 Removed / clarified content

- The orphan forward reference to a non-existent “Section 4.5 for confidence-threshold analysis” has been removed; the threshold rationale now sits in Section 3.7.2.
- The phrase *“professional agricultural standards”* in Section 4.5 has been rewritten as a description of the GUI’s controls.
- The hyperbolic phrases 'dramatic improvement', 'impressive 98.9 %', and 'a robust and efficient solution' — together with other similar soft-hype phrasing identified in a manuscript-wide pass — have been removed from the revised text.
- The unused **IEI** metric definition has been removed; the deployment-relevant efficiency information is now reported concretely as CPU Latency, FPS, and PPR across Tables 2, 3, and 4.

### 4.5 External depositions

- **Dataset.** The complete 9,591-image dataset has been deposited on figshare under DOI **10.6084/m9.figshare.32253357** (CC BY 4.0; <https://doi.org/10.6084/m9.figshare.32253357>). The Data Availability Statement in the submission system has been updated accordingly.
- **Source code.** The reference implementation — model definition, training / evaluation / inference scripts, original Kaggle notebook, trained checkpoint, and the architectural figure — is publicly released on GitHub at <https://github.com/lvxiangfly/tea-disease-detection> (release tag v1.0.0, GNU Affero General Public License v3.0).

## 5. Closing

We thank the academic editor and both reviewers for the time and care they have given to this submission. The combined feedback has improved the manuscript — particularly in tightening the comparative claims around the retrained baseline, in formalising the *Spatial-Aware* and *Path-Decoupling* contributions, and in surfacing the deployment-relevant inference latency as a primary axis of evaluation. We hope the revised version satisfactorily addresses every point raised and remain happy to provide additional clarification or further revisions on any point that remains open.

Sincerely,

**ChengLei Song** (corresponding author) *on behalf of Xiang Lyu and Yue Yu*

School of Management, Xinyang Agriculture and Forestry University Xinyang 464000, China 2015220006@xyafu.edu.cn
