# Supplementary material for: Spatial-aware lightweight network for real-time tea disease detection: A coordinate attention-enhanced YOLOv8n approach with path-decoupling strategy
Source: PLoS One. 2026 Jul 24;21(7):e0354583. doi: 10.1371/journal.pone.0354583 (PMC13399476; doi:10.1371/journal.pone.0354583)
Supplement: Peer review history

# Peer review: Decision Letter 0

**DOI:** 10.1371/journal.pone.0354583.r001

**Authors:** Xiaowei Li

1 May 2026

PONE-D-26-12938

Spatial-Aware Lightweight Network for Real-time Tea Disease Detection: A Coordinate Attention-Enhanced YOLOv8n Approach with Path-Decoupling Strategy

PLOS One

Dear Dr. Song,

Thank you for submitting your manuscript to PLOS ONE. After careful consideration, we feel that it has merit but does not fully meet PLOS ONE’s publication criteria as it currently stands. Therefore, we invite you to submit a revised version of the manuscript that addresses the points raised during the review process.

==============================

The reviewers have provided some necessary revision suggestions for your manuscript,  please revise the manuscript in accordance with their comments. A decision regarding whether to send the manuscript for a second round of review will subsequently be made based on the revisions you submit.

==============================

Please submit your revised manuscript by Jun 15 2026 11:59PM. If you will need more time than this to complete your revisions, please reply to this message or contact the journal office at plosone@plos.org. When you're ready to submit your revision, log on to https://www.editorialmanager.com/pone/ and select the 'Submissions Needing Revision' folder to locate your manuscript file.

Please include the following items when submitting your revised manuscript:

- A letter that responds to each point raised by the academic editor and reviewer(s). You should upload this letter as a separate file labeled 'Response to Reviewers'.
- A marked-up copy of your manuscript that highlights changes made to the original version. You should upload this as a separate file labeled 'Revised Manuscript with Track Changes'.
- An unmarked version of your revised paper without tracked changes. You should upload this as a separate file labeled 'Manuscript'.

If you would like to make changes to your financial disclosure, please include your updated statement in your cover letter. Guidelines for resubmitting your figure files are available below the reviewer comments at the end of this letter.

If applicable, we recommend that you deposit your laboratory protocols in protocols.io to enhance the reproducibility of your results. Protocols.io assigns your protocol its own identifier (DOI) so that it can be cited independently in the future. For instructions see: https://journals.plos.org/plosone/s/submission-guidelines#loc-laboratory-protocols. Additionally, PLOS ONE offers an option for publishing peer-reviewed Lab Protocol articles, which describe protocols hosted on protocols.io. Read more information on sharing protocols at https://plos.org/protocols?utm_medium=editorial-email&utm_source=authorletters&utm_campaign=protocols.

As the corresponding author, your ORCID iD is verified in the submission system and will appear in the published article. PLOS supports the use of ORCID, and we encourage all coauthors to register for an ORCID iD and use it as well. Please encourage your coauthors to verify their ORCID iD within the submission system before final acceptance, as unverified ORCID iDs will not appear in the published article. *Only*  the individual author can complete the verification step; PLOS staff *cannot*  verify ORCID iDs on behalf of authors.

We look forward to receiving your revised manuscript.

Kind regards,

Xiaowei Li

Academic Editor

PLOS One

Journal Requirements:

When submitting your revision, we need you to address these additional requirements.

1. Please ensure that your manuscript meets PLOS ONE's style requirements, including those for file naming. The PLOS ONE style templates can be found at https://journals.plos.org/plosone/s/file?id=wjVg/PLOSOne_formatting_sample_main_body.pdf and https://journals.plos.org/plosone/s/file?id=ba62/PLOSOne_formatting_sample_title_authors_affiliations.pdf

2. Please note that PLOS One has specific guidelines on code sharing for submissions in which author-generated code underpins the findings in the manuscript. In these cases, we expect all author-generated code to be made available without restrictions upon publication of the work. Please review our guidelines at https://journals.plos.org/plosone/s/materials-and-software-sharing#loc-sharing-code and ensure that your code is shared in a way that follows best practice and facilitates reproducibility and reuse.

3. Thank you for uploading your study's underlying data set. Unfortunately, the repository you have noted in your Data Availability statement does not qualify as an acceptable data repository according to PLOS's standards.

At this time, please upload the minimal data set necessary to replicate your study's findings to a stable, public repository (such as figshare or Dryad) and provide us with the relevant URLs, DOIs, or accession numbers that may be used to access these data. For a list of recommended repositories and additional information on PLOS standards for data deposition, please see https://journals.plos.org/plosone/s/recommended-repositories.

4. PLOS requires an ORCID iD for the corresponding author in Editorial Manager on papers submitted after December 6th, 2016. Please ensure that you have an ORCID iD and that it is validated in Editorial Manager. To do this, go to ‘Update my Information’ (in the upper left-hand corner of the main menu), and click on the Fetch/Validate link next to the ORCID field. This will take you to the ORCID site and allow you to create a new iD or authenticate a pre-existing iD in Editorial Manager.

5. We note that Figures 2, 3, 6, 8, and 9 in your submission contain images which may be copyrighted. All PLOS content is published under the Creative Commons Attribution License (CC BY 4.0), which means that the manuscript, images, and Supporting Information files will be freely available online, and any third party is permitted to access, download, copy, distribute, and use these materials in any way, even commercially, with proper attribution. For more information, see our copyright guidelines: http://journals.plos.org/plosone/s/licenses-and-copyright.

We require you to either (1) present written permission from the copyright holder to publish these figures specifically under the CC BY 4.0 license, or (2) remove the figures from your submission:

1. You may seek permission from the original copyright holder of Figures 2, 3, 6, 8, and 9 to publish the content specifically under the CC BY 4.0 license.

We recommend that you contact the original copyright holder with the Content Permission Form (http://journals.plos.org/plosone/s/file?id=7c09/content-permission-form.pdf) and the following text:

“I request permission for the open-access journal PLOS ONE to publish XXX under the Creative Commons Attribution License (CCAL) CC BY 4.0 (http://creativecommons.org/licenses/by/4.0/). Please be aware that this license allows unrestricted use and distribution, even commercially, by third parties. Please reply and provide explicit written permission to publish XXX under a CC BY license and complete the attached form.”

Please upload the completed Content Permission Form or other proof of granted permissions as an "Other" file with your submission.

In the figure caption of the copyrighted figure, please include the following text: “Reprinted from [ref] under a CC BY license, with permission from [name of publisher], original copyright [original copyright year].”

2. If you are unable to obtain permission from the original copyright holder to publish these figures under the CC BY 4.0 license or if the copyright holder’s requirements are incompatible with the CC BY 4.0 license, please either i) remove the figure or ii) supply a replacement figure that complies with the CC BY 4.0 license. Please check copyright information on all replacement figures and update the figure caption with source information. If applicable, please specify in the figure caption text when a figure is similar but not identical to the original image and is therefore for illustrative purposes only.

6. We note you have included a table to which you do not refer in the text of your manuscript. Please ensure that you refer to Table 2 in your text; if accepted, production will need this reference to link the reader to the Table.

7. Please include a copy of Table 4.5 which you refer to in your text on page 15.

If the reviewer comments include a recommendation to cite specific previously published works, please review and evaluate these publications to determine whether they are relevant and should be cited. There is no requirement to cite these works unless the editor has indicated otherwise.

[Note: HTML markup is below. Please do not edit.]

Reviewers' comments:

Reviewer's Responses to Questions

**Comments to the Author**

1. Is the manuscript technically sound, and do the data support the conclusions?

The manuscript must describe a technically sound piece of scientific research with data that supports the conclusions. Experiments must have been conducted rigorously, with appropriate controls, replication, and sample sizes. The conclusions must be drawn appropriately based on the data presented.

Reviewer #1: Yes

Reviewer #2: Partly

**********

2. Has the statistical analysis been performed appropriately and rigorously?

Reviewer #1: Yes

Reviewer #2: No

**********

3. Have the authors made all data underlying the findings in their manuscript fully available?

The PLOS Data policy requires authors to make all data underlying the findings described in their manuscript fully available without restriction, with rare exception (please refer to the Data Availability Statement in the manuscript PDF file). The data should be provided as part of the manuscript or its supporting information, or deposited to a public repository. For example, in addition to summary statistics, the data points behind means, medians and variance measures should be available. If there are restrictions on publicly sharing data—e.g. participant privacy or use of data from a third party—those must be specified.

Reviewer #1: Yes

Reviewer #2: No

**********

4. Is the manuscript presented in an intelligible fashion and written in standard English?

PLOS ONE does not copyedit accepted manuscripts, so the language in submitted articles must be clear, correct, and unambiguous. Any typographical or grammatical errors should be corrected at revision, so please note any specific errors here.

Reviewer #1: Yes

Reviewer #2: Yes

**********

5. Review Comments to the Author

Please use the space provided to explain your answers to the questions above. You may also include additional comments for the author, including concerns about dual publication, research ethics, or publication ethics. (Please upload your review as an attachment if it exceeds 20,000 characters)

Reviewer #1: The research is interesting; however, it would benefit from the inclusion of more relevant literature to ensure that the context is comprehensively covered. It is also recommended to include a summary table at the end of the literature review to compare existing approaches, which would help to clearly position the proposed method within the current body of research.

The paper introduces multiple enhancements (coordinate attention, path-decoupling, lightweight design), but it is not entirely clear how each component individually contributes to performance. Consider adding an ablation study to isolate the impact of each module.

The term “spatial-aware” is used prominently in the title, but its technical definition and implementation are not sufficiently explained. Clarify how spatial awareness is modeled differently from standard YOLOv8 attention mechanisms.

The choice of enhancing YOLOv8n specifically should be better justified. Why is the nano version preferred over other variants (e.g., YOLOv8s/m)? Is the focus primarily on edge deployment or computational constraints?

The proposed path-decoupling strategy needs clearer explanation. A diagram or architectural comparison would help readers understand how it differs from conventional detection heads.

Since the work targets real-time tea disease detection, discuss:

Deployment feasibility on edge devices:

Performance under real-world conditions (e.g., varying weather, leaf overlap). Explain further on this.

Include a brief discussion on limitations of the study.

Therefore, it is recommended to conduct multiple experimental setups to further validate the robustness of the proposed method and potentially improve the overall results. This could include varying training configurations, testing under different conditions, or evaluating on additional datasets.

Reviewer #2: The paper proposes CA-YOLOv8n, a lightweight object detection model combining Coordinate Attention (CA) with a Path-Decoupling strategy on YOLOv8n for tea disease detection, targeting edge deployment. It appears that important sections of the papers were not included although references were made to such sections. For example: section 3.1.1 and section 4.5

Here are the comments on different aspects of the article

# Dataset (Section 3.1)

- No statistical distribution figure is present despite "3.1.1 Statistical Distribution" being listed as a subsection header with no content. This is a placeholder left unfilled.

- Class distribution of the dataset is not discussed, potentially ignoring any class imbalance issues. Eight classes across 9,591 images with no per-class breakdown is a significant omission.

- The dataset is sourced from "Tencent Developer Community" with no DOI, or verifiable link. Reproducibility is impossible without this.

# Experimental Design (Section 3)

- The confidence threshold of τ=0.25 is presented as a design choice, but its effect on precision is not rigorously quantified in a dedicated ablation — it is only briefly discussed in Section 3.6.2. Although the authors referred to Section 4.5 for relevant analysis, but the article do not contain any such section.

- No training hardware specification is provided (only inference hardware in Section 4.3.3). Training reproducibility is compromised.

- No cross-validation or repeated trial protocol is described. Results from a single train/test split are insufficient to establish generalizability claims.

# Comparative Analysis (Section 4.3)

- Table 1 compares only three models (YOLOv8n, YOLOv10n, CA-YOLOv8n). No comparison against other lightweight tea-specific models (e.g., YOLO-Tea from reference [28], Dise-Efficient from [19]) is made, despite these being directly cited in the related work.

- The 18.09% mAP improvement cited in Section 3.5.1 does not match the calculated difference from Table 1 (83.7% → 98.9% = 15.2 percentage points). Please explain the inconsistency in the reporting.

- When reporting computational efficiency, inference speed (FPS/ms latency), which is central to the edge deployment problem.

# Confusion Matrix (Section 4.2)

- The "disease" (other minor diseases) category shows no true positive values in the confusion matrix (Fig. 7) and should be discussed.

- False negative rates for Tea Mosquito bug (0.52) and Red Spider (0.27) are high and warrant deeper analysis.

# ECGR Metrics

- IEI metric was defined but never actually reported in any table.

# Deployment:

- as mentioned, the system interface was developed following "professional agricultural standards". Please specify which standards are you referring to

- the authors mentioned about deployment on the edge, please provide more hard ware/software details of the edge platform used.

# Language:

Writing is generally intelligible but contains hyperbolic phrasing inappropriate for scientific publication (e.g., "dramatic improvement," "impressive 98.9%," "a robust and efficient solution").

**********

6. PLOS authors have the option to publish the peer review history of their article (what does this mean?). If published, this will include your full peer review and any attached files.

If you choose “no”, your identity will remain anonymous but your review may still be made public.

**Do you want your identity to be public for this peer review?**  For information about this choice, including consent withdrawal, please see our Privacy Policy.

Reviewer #1: No

Reviewer #2: No

**********

[NOTE: If reviewer comments were submitted as an attachment file, they will be attached to this email and accessible via the submission site. Please log into your account, locate the manuscript record, and check for the action link "View Attachments". If this link does not appear, there are no attachment files.]

To ensure your figures meet our technical requirements, please review our figure guidelines: https://journals.plos.org/plosone/s/figures

You may also use PLOS’s free figure tool, NAAS, to help you prepare publication quality figures: https://journals.plos.org/plosone/s/figures#loc-tools-for-figure-preparation.

NAAS will assess whether your figures meet our technical requirements by comparing each figure against our figure specifications.

# Author comment: Author response to Decision Letter 1

**DOI:** 10.1371/journal.pone.0354583.r002

4 Jun 2026

Dear Dr. Xiaowei Li and the two anonymous Reviewers,

We thank the academic editor and both reviewers for the detailed, constructive comments on our submission. Each point has been addressed in the revised manuscript, and a marked-up copy with track changes has been uploaded alongside this letter. Where a comment is cross-referenced to a section, figure, or table, the section/figure/table number refers to the revised manuscript. Two new figures have been added during the revision — Fig 2 (the Path-Decoupling neck comparison requested by Reviewer #1, R1.5) and Fig 10 (the per-class diagnostic produced for the new ablation study, requested by Reviewer #1, R1.2). A new literature-summary Table 1 has also been added at the end of Section 2 (in response to Reviewer #1, R1.1), so the three pre-existing tables have been renumbered: Comparative performance → Table 2, Computational efficiency → Table 3, Module-level ablation → Table 4. All previously existing figures from old Fig 2 onward have been renumbered upward by one (old Fig 2 → new Fig 3, etc.).

The point-by-point responses are organised below in three sections: 1. Academic editor’s journal requirements (items 1–7) 2. Reviewer #1 (seven comments) 3. Reviewer #2 (comments grouped by section)

A summary list of every change made to the manuscript is provided at the end of this letter.

1. Academic Editor — Journal Requirements

Requirement 1. PLOS ONE style template

“Please ensure that your manuscript meets PLOS ONE’s style requirements, including those for file naming.”

Authors’ response: The manuscript has been reformatted to follow the PLOS ONE style template (main body and title/authors/affiliations samples linked in the decision letter). Section headings, paragraph spacing, figure/table captions, and reference formatting now conform to the PLOS ONE guidelines.

Requirement 2. Author-generated code must be openly available

“Please note that PLOS One has specific guidelines on code sharing for submissions in which author-generated code underpins the findings in the manuscript. We expect all author-generated code to be made available without restrictions upon publication of the work.”

Authors’ response: A reference implementation has been released publicly on GitHub under the GNU Affero General Public License v3.0 (the licence required by the Ultralytics YOLOv8 framework on which the work builds):

Repository: https://github.com/lvxiangfly/tea-disease-detection Release tag: v1.0.0 Direct link to the release snapshot: https://github.com/lvxiangfly/tea-disease-detection/releases/tag/v1.0.0

The release contains the Coordinate-Attention module, the CA-YOLOv8n model YAML, the original Kaggle training notebook that produced the checkpoint reported in this paper, portable training/evaluation/inference scripts, the CPU latency benchmark script used for Table 2, and the final trained weight file.

Requirement 3. Dataset must be deposited in a PLOS-acceptable repository

“Unfortunately, the repository you have noted in your Data Availability statement does not qualify as an acceptable data repository according to PLOS’s standards. At this time, please upload the minimal data set necessary to replicate your study’s findings to a stable, public repository (such as figshare or Dryad).”

Authors’ response: The complete 9,591-image tea-leaf disease dataset has been deposited on figshare under DOI:

DOI: 10.6084/m9.figshare.32253357 URL: https://doi.org/10.6084/m9.figshare.32253357 Licence: CC BY 4.0

Requirement 4. ORCID for corresponding author validated in Editorial Manager

“PLOS requires an ORCID iD for the corresponding author in Editorial Manager on papers submitted after December 6th, 2016.”

Authors’ response: An ORCID iD for the corresponding author has been registered at orcid.org and will be validated via the Fetch/Validate link in Editorial Manager prior to resubmission.

Requirement 5. Figure copyright check (Figures 2, 3, 6, 8, 9 in the original submission)

“We note that Figures 2, 3, 6, 8, and 9 in your submission contain images which may be copyrighted… We require you to either (1) present written permission from the copyright holder to publish these figures specifically under the CC BY 4.0 license, or (2) remove the figures from your submission.”

Authors’ response: After re-examining all flagged figures, we clarify the situation as follows. The five figures cited in the editor’s note (using the original submission’s numbering) correspond to:

Original submission # Revised manuscript # Caption (short) Authorship

Fig 2 Fig 3 Architectural framework of the tea-disease identification system Original — drawn by the authors

Fig 3 Fig 4 Detailed inference pipeline and dynamic threshold decision-making logic Original — drawn by the authors

Fig 6 Fig 7 Batch Samples Composed of dataset images (see below)

Fig 8 Fig 9 Qualitative comparison of detection results Composed of dataset images (see below)

Fig 9 Fig 11 The GUI of the Tea Disease Identification System Original — author’s software screenshot

Originally created by the authors (no third-party content reused). Figs 1,2, 3, 4, 5,6,8, 10, and 11 of the revised manuscript — comprising the architectural diagrams, the confusion matrix, the per-class diagnostic, and the GUI screenshot — were produced entirely by the authors using draw.io, matplotlib, and our own GUI application, so the CC BY 4.0 licence applies without restriction.

Drawn from the authors' figshare dataset. Figs 7 and 9 incorporate sample images from the tea-leaf disease dataset deposited at figshare DOI 10.6084/m9.figshare.32253357 (CC BY 4.0). Per the editorial office's latest request prior to peer review, we have now explicitly added the relevant attribution, DOI, and usage information (CC BY 4.0) directly into the captions of Fig 7 and Fig 9 in the revised manuscript.

Requirement 6. Table 2 must be referred to in the text

“We note you have included a table to which you do not refer in the text of your manuscript. Please ensure that you refer to Table 2 in your text.”

Authors’ response: The table the editor flagged (Analysis of computational efficiency and parameter utilization) — originally Table 2, now Table 3 in the revised manuscript after a new literature-summary Table 1 was added at the end of Section 2 — is now explicitly cited in Section 4.3.2 (Analysis of Computational Efficiency), which discusses each column of the table and links the parameter-efficiency ratio (PPR) of CA-YOLOv8n to the Path-Decoupling design.

Requirement 7. Missing Table 4.5 referenced on page 15

“Please include a copy of Table 4.5 which you refer to in your text on page 15.”

Authors’ response: The original reference to “Table 4.5” was a typographical placeholder that was no longer needed once the ablation analysis was consolidated. The reference has been removed, and the ablation results are now presented in Table 4 (Module-level ablation on the independent test set) of the revised manuscript and complemented by Fig 10 (Per-class diagnostic performance of the four ablation variants). No further “Table 4.5” reference appears anywhere in the revised text.

2. Reviewer #1

R1.1. Additional literature and a literature-review summary table

“The research is interesting; however, it would benefit from the inclusion of more relevant literature to ensure that the context is comprehensively covered. It is also recommended to include a summary table at the end of the literature review to compare existing approaches, which would help to clearly position the proposed method within the current body of research.”

Authors’ response: The Related Work section has been expanded along three lines. (i) Additional references covering attention-based pest/disease detection, lightweight YOLO variants for agricultural deployment, and tea-domain works have been added. (ii) A new comparative-summary Table 1 (Comparison of existing models for agricultural disease detection) has been inserted at the end of Section 2; it compares five representative recent models across four axes (base architecture, key enhancements, and limitations) — YOLO-Tea (Xue et al., 2023) , YOLOv8-RCAA (Wang et al., 2024) , MobileNet-CA-YOLO (Jia et al., 2023) , Dise-Efficient (Guan et al., 2023) , and AX-RetinaNet (Bao et al., 2022) — three of which (YOLO-Tea, YOLOv8-RCAA, AX-RetinaNet) are explicitly tea-disease detectors, providing direct in-domain positioning. (iii) The narrative in Section 2 now references each row of Table 1 and articulates how the proposed CA-YOLOv8n is positioned relative to these works (notably, Coordinate Attention is coupled with neck-side Path-Decoupling rather than inserted into the backbone as in MobileNet-CA-YOLO). Because Table 1 has been inserted at the start of the numbered table sequence, the three pre-existing tables have been renumbered accordingly (Comparative performance → Table 2; Computational efficiency → Table 3; Module-level ablation → Table 4), and all in-text and Response-letter references below use the revised numbering.

R1.2. Ablation study isolating each module

“The paper introduces multiple enhancements (coordinate attention, path-decoupling, lightweight design), but it is not entirely clear how each component individually contributes to performance. Consider adding an ablation study to isolate the impact of each module.”

Authors’ response: A controlled 2 × 2 ablation has been added in Section 4.4 (Ablation Study) of the revised manuscript. The four variants — Baseline (YOLOv8n), + CA only, + PD only, and CA-YOLOv8n (Full) — share an identical training protocol and are reported together in Table 4 (parameters, GFLOPs, mAP@50, mAP@50-95, Precision, Recall, Latency, FPS) and in Fig 10 (per-class Precision / Recall / mAP@50 / mAP@50-95). The ablation isolates the contributions of each component: the +CA-only variant raises mAP@50 from 97.11 % to 98.74 % while leaving the parameter count essentially unchanged (3.01 M → 3.02 M); the +PD-only variant reduces parameters and GFLOPs by 33 % and 24 % respectively (3.01 M → 2.03 M, 8.20 → 6.21 GFLOPs) and cuts CPU latency from 63.62 ms to 48.85 ms; combining the two recovers the precision gain while preserving the lightweight footprint of PD (2.03 M / 6.22 GFLOPs / 51.39 ms / mAP@50 = 98.89 %, mAP@50-95 = 76.26 %). The complementary failure modes the two modules address (CA improves Precision, PD improves Recall) are discussed in Section 4.4.

R1.3. Technical definition of “Spatial-Aware”

“The term ‘spatial-aware’ is used prominently in the title, but its technical definition and implementation are not sufficiently explained. Clarify how spatial awareness is modeled differently from standard YOLOv8 attention mechanisms.”

Authors’ response: Two paragraphs have been added at the opening of Section 3.3 (Coordinate Attention and Architectural Compensation) to define the term and contrast it with the channel-only attention typically paired with YOLOv8. Briefly: standard channel-attention modules such as Squeeze-and-Excitation (Hu et al., 2018) collapse every spatial location of a channel into a single scalar via global average pooling, so the resulting gating signal is position-agnostic within a channel. Coordinate Attention replaces that 2D global pooling with two complementary 1D pooling kernels of size (H, 1) and (1, W), which aggregate features along the vertical and horizontal axes separately; the direction-aware descriptors are concatenated, passed through a shared 1 × 1 convolution, and split back into a height-wise and a width-wise attention map, each broadcast over its orthogonal axis when modulating the input. The recalibration weights therefore vary across spatial coordinates rather than being shared within a channel — this row- and column-conditioned gating is the precise sense in which the proposed network is Spatial-Aware, and it is what distinguishes Coordinate Attention from SE and from the position-agnostic attention designs commonly inserted into YOLOv8. Section 3.3 then formalises the two stages of CA (Coordinate Information Embedding in Section 3.3.1 and Coordinate Attention Generation in Section 3.3.2) before integrating its output under the Path-Decoupling design illustrated in Fig 2.

R1.4. Justification for choosing YOLOv8n (nano) over s / m variants

“The choice of enhancing YOLOv8n specifically should be better justified. Why is the nano version preferred over other variants (e.g., YOLOv8s/m)? Is the focus primarily on edge deployment or computational constraints?”

Authors’ response: The choice is dictated by the target deployment scenario stated in the Introduction (Section 1) and reinforced in Section 4.3 (Performance Comparison): a CPU-only edge platform represented in this study by an Intel Core i5-13500H. The nano variant is the only scale of the YOLOv8 family whose parameter footprint (~3 M) and FLOP budget (~8 GFLOPs) keep single-image CPU latency within the sub-100 ms range that the agricultural inspection workflow targeted in this paper requires. YOLOv8s (~11 M parameters, ~28 GFLOPs) and YOLOv8m (~26 M, ~79 GFLOPs) would multiply CPU latency by roughly 4 × and 12 × respectively.

R1.5. Diagram / architectural comparison of the Path-Decoupling strategy

“The proposed path-decoupling strategy needs clearer explanation. A diagram or architectural comparison would help readers understand how it differs from conventional detection heads.”

Authors’ response: A side-by-side architectural comparison has been added as Fig 2 in the revised manuscript. Panel (a) depicts the standard PAN-FPN neck used by YOLOv8n — 12 successive operations (2 Upsample + 4 Concat + 4 C2f + 2 Conv-down) sitting between the backbone outputs (P3, P4, P5) and the three detection heads. Panel (b) depicts the proposed Streamlined-Neck: the entire PAN-FPN neck is removed, P3 (layer 4) and P4 (layer 6) are routed directly from the backbone into the detection heads, and only a single Coordinate-Attention block is inserted on the deepest backbone feature P5. The figure caption quantifies the cost differential (≈ 0.98 M parameters and ≈ 1.98 GFLOPs removed; 12 fusion layers replaced by 1 attention layer), and the Path-Decoupling rationale is now formalised in Section 3.3.3 (Feature Recalibration under Path-Decoupling). The matplotlib script used to render Fig 2 is included in the public code release (docs/architecture.png / .pdf) for full reproducibility.

R1.6. Edge deployment feasibility, real-world conditions, and Limitations

“Since the work targets real-time tea disease detection, discuss: Deployment feasibility on edge devices; Performance under real-world conditions (e.g., varying weather, leaf overlap). Explain further on this. Include a brief discussion on limitations of the study.”

Authors’ response: All three sub-points have been addressed in the revised manuscript.

Edge deployment feasibility. The subsection Performance Advantages of Lightweight Deployment (now part of the System Implementation and Deployment block in Section 3) lists the exact deployment platform (Intel Core i5-13500H CPU, single thread, batch = 1, FP32, 640 × 640) and reports the achieved end-to-end inference latency (51.39 ms ≈ 19.5 FPS). Section 4.3 (Performance Comparison) further benchmarks CA-YOLOv8n against YOLOv8n and YOLOv10n on the same hardware (51.39 ms vs. 63.62 ms and 179.00 ms; see Table 2), confirming that the proposed design meets the sub-100 ms real-time budget on commodity CPU hardware without GPU acceleration.

Performance under real-world conditions. Fig 9 (Qualitative comparison of detection results between baseline YOLOv8n and the proposed CA-YOLOv8n under complex tea plantation environments) presents detection examples that include occluded leaves, overlapping foliage, and varying illumination. The accompanying narrative in Section 4.3.1 walks through several representative cases where the baseline either misses heavily overlapping lesions or misclassifies them, and shows the corresponding CA-YOLOv8n predictions. The class-imbalance discussion newly added to Section 3.1.1 (Statistical Distribution) and the confusion-matrix analysis in Section 4.2 further quantify the failure modes that remain under real-world distribution shift.

Limitations. A dedicated Section 5.2 (Limitations) has been added to the revised manuscript. It explicitly acknowle

Attachment Submitted filename: RESPONSE_TO_REVIEWERS.docx

# Peer review: Decision Letter 1

**DOI:** 10.1371/journal.pone.0354583.r003

**Authors:** Xiaowei Li

9 Jul 2026

Spatial-Aware Lightweight Network for Real-time Tea Disease Detection: A Coordinate Attention-Enhanced YOLOv8n Approach with Path-Decoupling Strategy

PONE-D-26-12938R1

Dear Dr. Song,

We’re pleased to inform you that your manuscript has been judged scientifically suitable for publication and will be formally accepted for publication once it meets all outstanding technical requirements.

Within one week, you’ll receive an e-mail detailing the required amendments. When these have been addressed, you’ll receive a formal acceptance letter and your manuscript will be scheduled for publication.

An invoice will be generated when your article is formally accepted. Please note, if your institution has a publishing partnership with PLOS and your article meets the relevant criteria, all or part of your publication costs will be covered. Please make sure your user information is up-to-date by logging into Editorial Manager at Editorial Manager® and clicking the ‘Update My Information' link at the top of the page. For questions related to billing, please contact billing support.

If your institution or institutions have a press office, please notify them about your upcoming paper to help maximize its impact. If they’ll be preparing press materials, please inform our press team as soon as possible -- no later than 48 hours after receiving the formal acceptance. Your manuscript will remain under strict press embargo until 2 pm Eastern Time on the date of publication. For more information, please contact onepress@plos.org.

Kind regards,

Xiaowei Li

Academic Editor

PLOS One

Reviewers' comments:

Reviewer's Responses to Questions

**Comments to the Author**

1. If the authors have adequately addressed your comments raised in a previous round of review and you feel that this manuscript is now acceptable for publication, you may indicate that here to bypass the “Comments to the Author” section, enter your conflict of interest statement in the “Confidential to Editor” section, and submit your "Accept" recommendation.

Reviewer #2: All comments have been addressed

**********

2. Is the manuscript technically sound, and do the data support the conclusions?

The manuscript must describe a technically sound piece of scientific research with data that supports the conclusions. Experiments must have been conducted rigorously, with appropriate controls, replication, and sample sizes. The conclusions must be drawn appropriately based on the data presented.

Reviewer #2: Yes

**********

3. Has the statistical analysis been performed appropriately and rigorously?

Reviewer #2: Yes

**********

4. Have the authors made all data underlying the findings in their manuscript fully available?

The PLOS Data policy requires authors to make all data underlying the findings described in their manuscript fully available without restriction, with rare exception (please refer to the Data Availability Statement in the manuscript PDF file). The data should be provided as part of the manuscript or its supporting information, or deposited to a public repository. For example, in addition to summary statistics, the data points behind means, medians and variance measures should be available. If there are restrictions on publicly sharing data—e.g. participant privacy or use of data from a third party—those must be specified.

Reviewer #2: Yes

**********

5. Is the manuscript presented in an intelligible fashion and written in standard English?

PLOS ONE does not copyedit accepted manuscripts, so the language in submitted articles must be clear, correct, and unambiguous. Any typographical or grammatical errors should be corrected at revision, so please note any specific errors here.

Reviewer #2: Yes

**********

6. Review Comments to the Author

Please use the space provided to explain your answers to the questions above. You may also include additional comments for the author, including concerns about dual publication, research ethics, or publication ethics. (Please upload your review as an attachment if it exceeds 20,000 characters)

Reviewer #2: The manuscript appears to be mostly generated using generative AI but the author did not disclose anything explicitly in this regard

**********

7. PLOS authors have the option to publish the peer review history of their article (what does this mean?). If published, this will include your full peer review and any attached files.

If you choose “no”, your identity will remain anonymous but your review may still be made public.

**Do you want your identity to be public for this peer review?**  For information about this choice, including consent withdrawal, please see our Privacy Policy.

Reviewer #2: No

**********

# Editor report: Acceptance letter

**DOI:** 10.1371/journal.pone.0354583.r004

**Authors:** Xiaowei Li

PONE-D-26-12938R1

PLOS One

Dear Dr. Song,

I'm pleased to inform you that your manuscript has been deemed suitable for publication in PLOS One. Congratulations! Your manuscript is now being handed over to our production team.

At this stage, our production department will prepare your paper for publication. This includes ensuring the following:

* All references, tables, and figures are properly cited

* All relevant supporting information is included in the manuscript submission,

* There are no issues that prevent the paper from being properly typeset

You will receive further instructions from the production team, including instructions on how to review your proof when it is ready. Please keep in mind that we are working through a large volume of accepted articles, so please give us a few days to review your paper and let you know the next and final steps.

Lastly, if your institution or institutions have a press office, please let them know about your upcoming paper now to help maximize its impact. If they'll be preparing press materials, please inform our press team within the next 48 hours. Your manuscript will remain under strict press embargo until 2 pm Eastern Time on the date of publication. For more information, please contact onepress@plos.org.

You will receive an invoice from PLOS for your publication fee after your manuscript has reached the completed accept phase. If you receive an email requesting payment before acceptance or for any other service, this may be a phishing scheme. Learn how to identify phishing emails and protect your accounts at https://explore.plos.org/phishing.

If we can help with anything else, please email us at customercare@plos.org.

Thank you for submitting your work to PLOS ONE and supporting open access.

Kind regards,

PLOS ONE Editorial Office Staff

on behalf of

Dr. Xiaowei Li

Academic Editor

PLOS One
